# Supplementary material for: Semisynthetic Sesquiterpene Lactones Generated by the Sensibility of Glaucolide B to Lewis and Brønsted–Lowry Acids and Bases: Cytotoxicity and Anti-Inflammatory Activities
Source: Molecules. 2023 Jan 27;28(3):1243. doi: 10.3390/molecules28031243 (PMC9921329; doi:10.3390/molecules28031243)

Supplementary Materials Contents:

Spectroscopic data for semisynthetic derivative **2**……………………………..……..………..…...……2
Spectroscopic data for semisynthetic derivative **3**………..………………………...…………...……5
Spectroscopic data for semisynthetic derivative **4**…………………………..………....………..……..…8
Spectroscopic data for semisynthetic derivative **5**………..………..……..……………....………..……11
Spectroscopic data for semisynthetic derivative **6**………..……………..……..……......………..……14
Spectroscopic data for semisynthetic derivative **7**………..……………..………..…………..…..……17
Spectroscopic data for semisynthetic derivative **8**………..……………..……......………..…..……20
Spectroscopic data for semisynthetic derivative **9**………..……………...………..………………...……23
Spectroscopic data for semisynthetic derivative **7a**…..…..……………..………..………..…..……26
Spectroscopic data for semisynthetic derivative **8a**…..…..……………..………......…………...……29
Spectroscopic data for semisynthetic derivative **9a**…..…..……………....………......……..………..…32
Spectroscopic data for semisynthetic derivative **7b**…..…..……………..………........…..…..……35
Spectroscopic data for semisynthetic derivative **7c**…..…..……………..………......……...…..……38
Spectroscopic data for semisynthetic derivative **7c.1**..…..……………..………......………..………41
Cell viability evaluation for macrophage treated with SLs **1-12**……...……………..……..…..……44

Figure S1 – ^{1}H NMR spectrum (300 MHz, $CDCl_3$, 295 K) of semisynthetic derivative **2**.

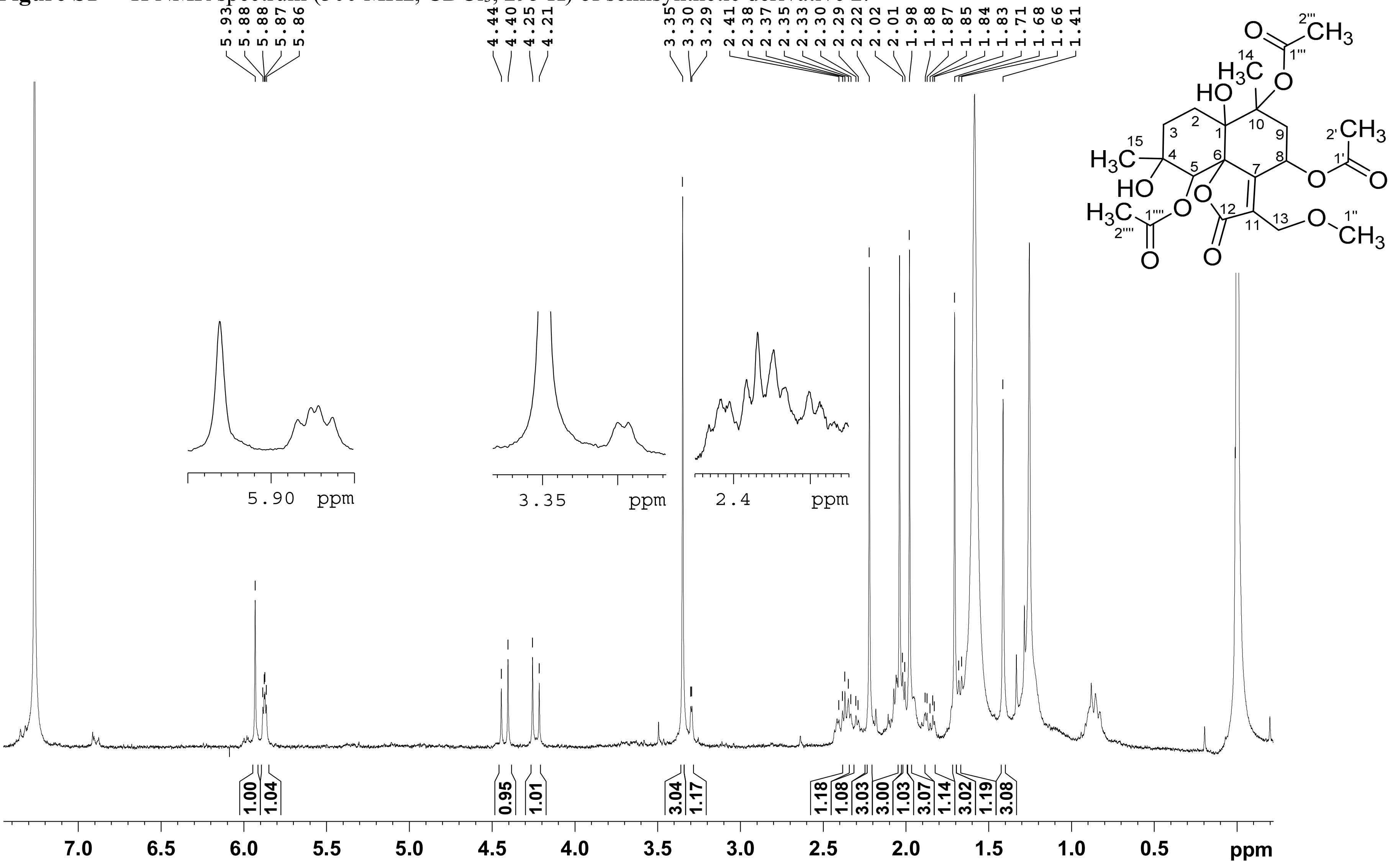

Chemical shift, in ppm (referenced to TMS - δ 0.00).

Figure S2 – HSQC correlation map ($CDCl_3$, 295 K) of semisynthetic derivative **2**.

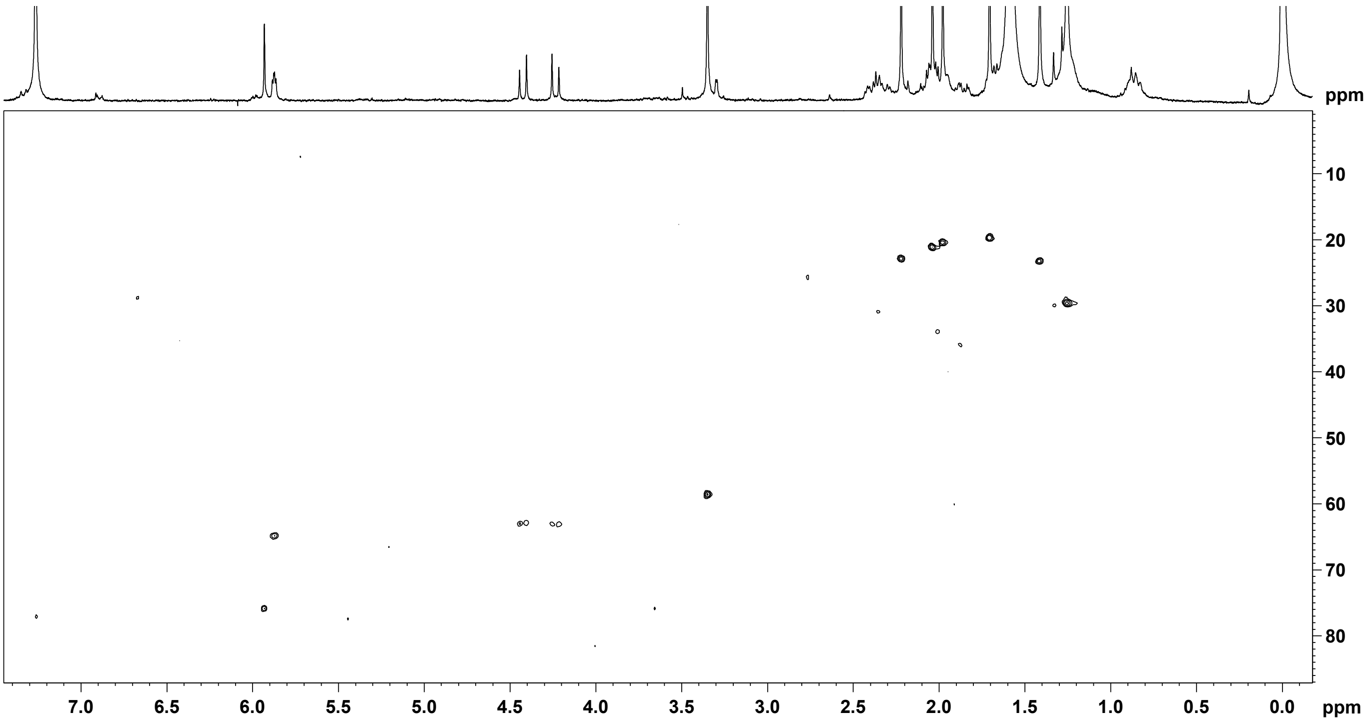

Chemical shift, in ppm (referenced to TMS - δ 0.00).

Figure S3 – HMBC correlation map ($CDCl_3$, 295 K) of semisynthetic derivative **2**.

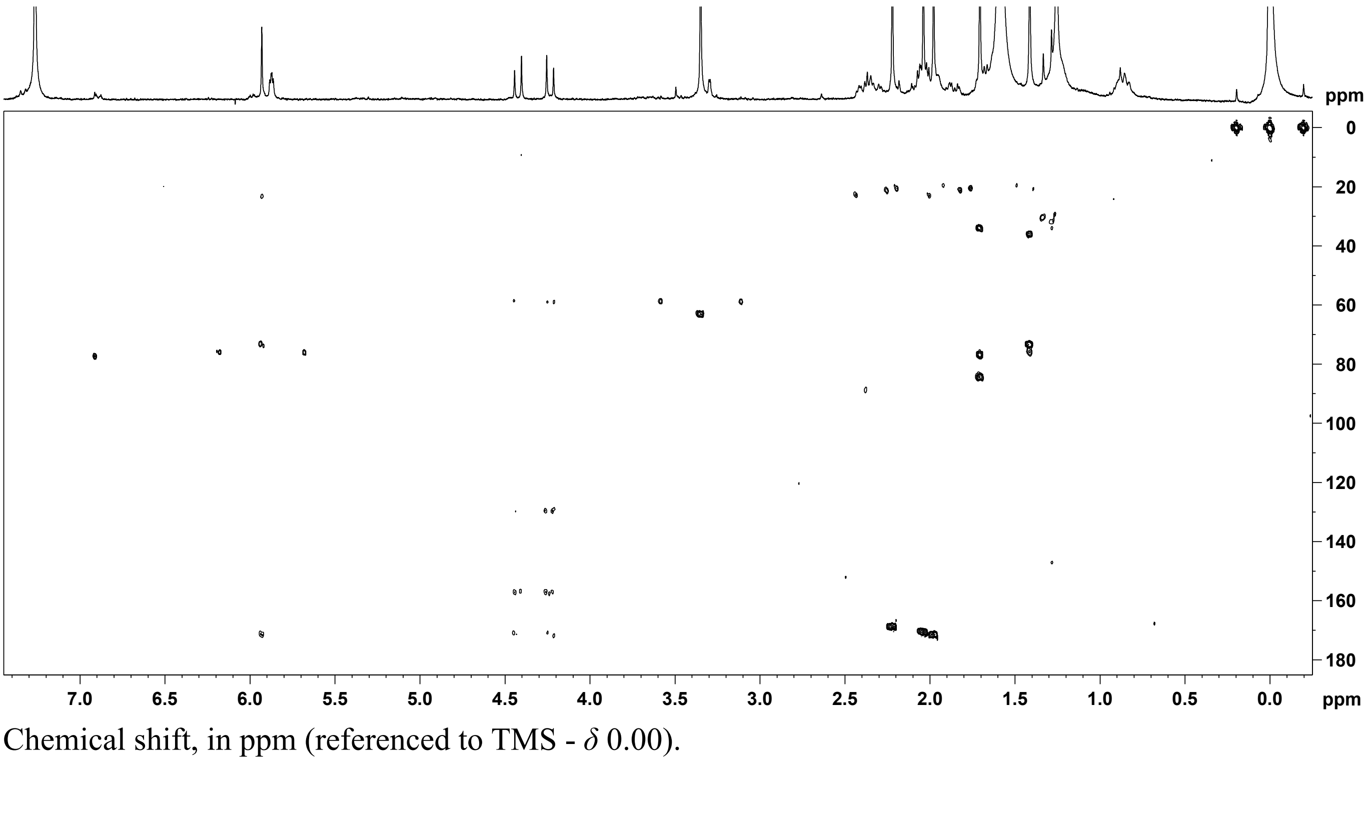

Chemical shift, in ppm (referenced to TMS - δ 0.00).

Figure S4 – HRESIMS *full scan* (100-1200 Da) spectrum of semisynthetic derivative **2**.

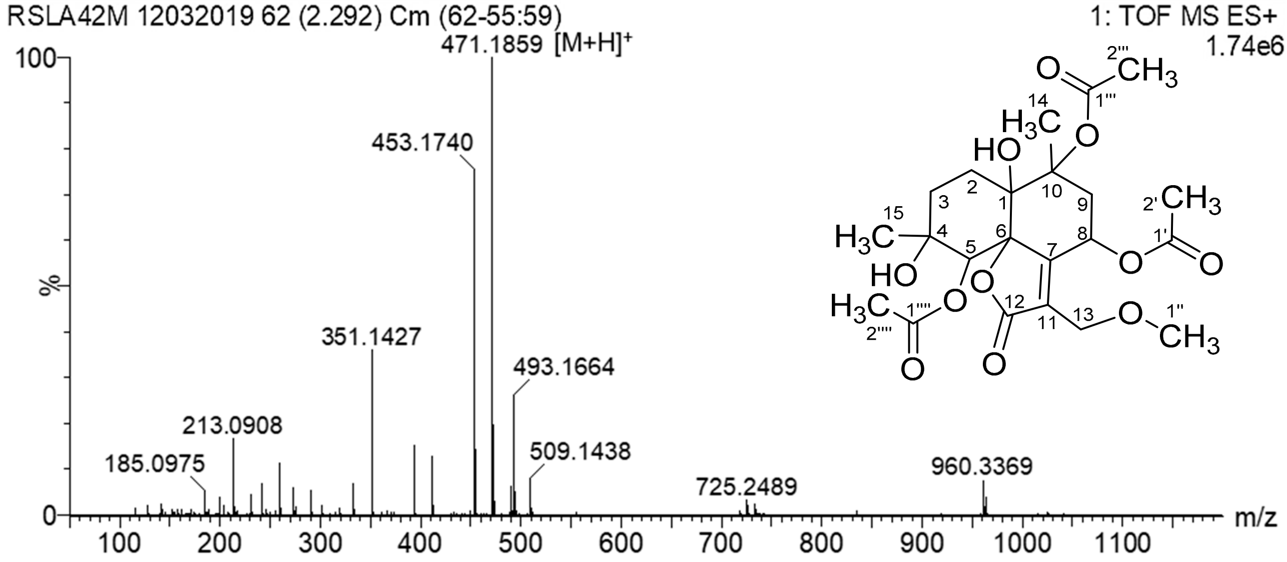

HRESIMS spectrum in positive mode achieved from UPLC-MS analysis.

Figure S5 – UV spectrum (200-400 nm) of semisynthetic derivative **2**.

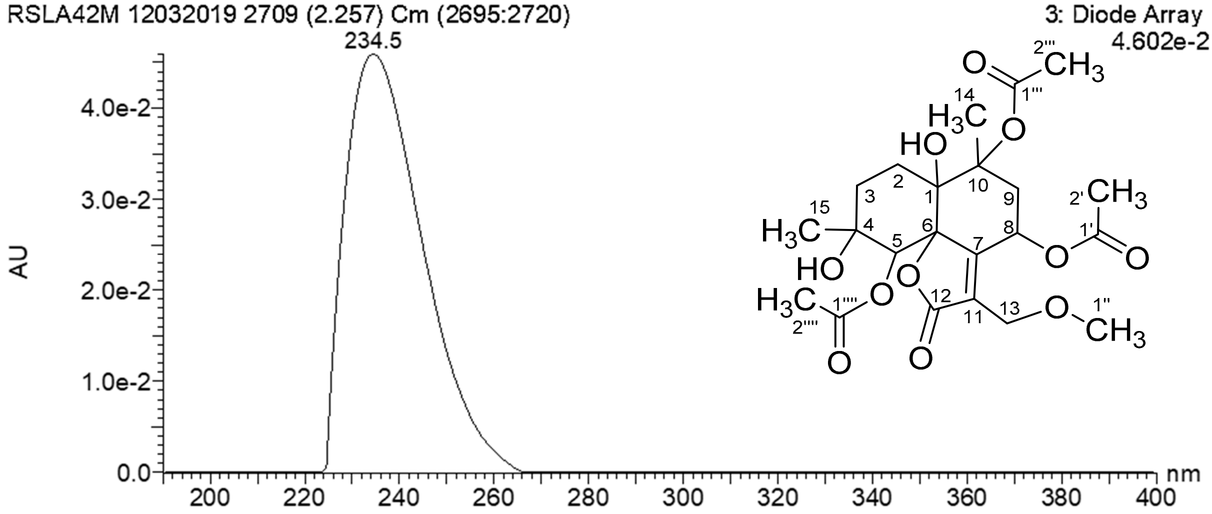

UV spectrum achieved from UPLC-PDA-MS analysis.

Figure S6 – ^{1}H NMR spectrum (300 MHz, $CDCl_3$, 295 K) of semisynthetic derivative **3**.

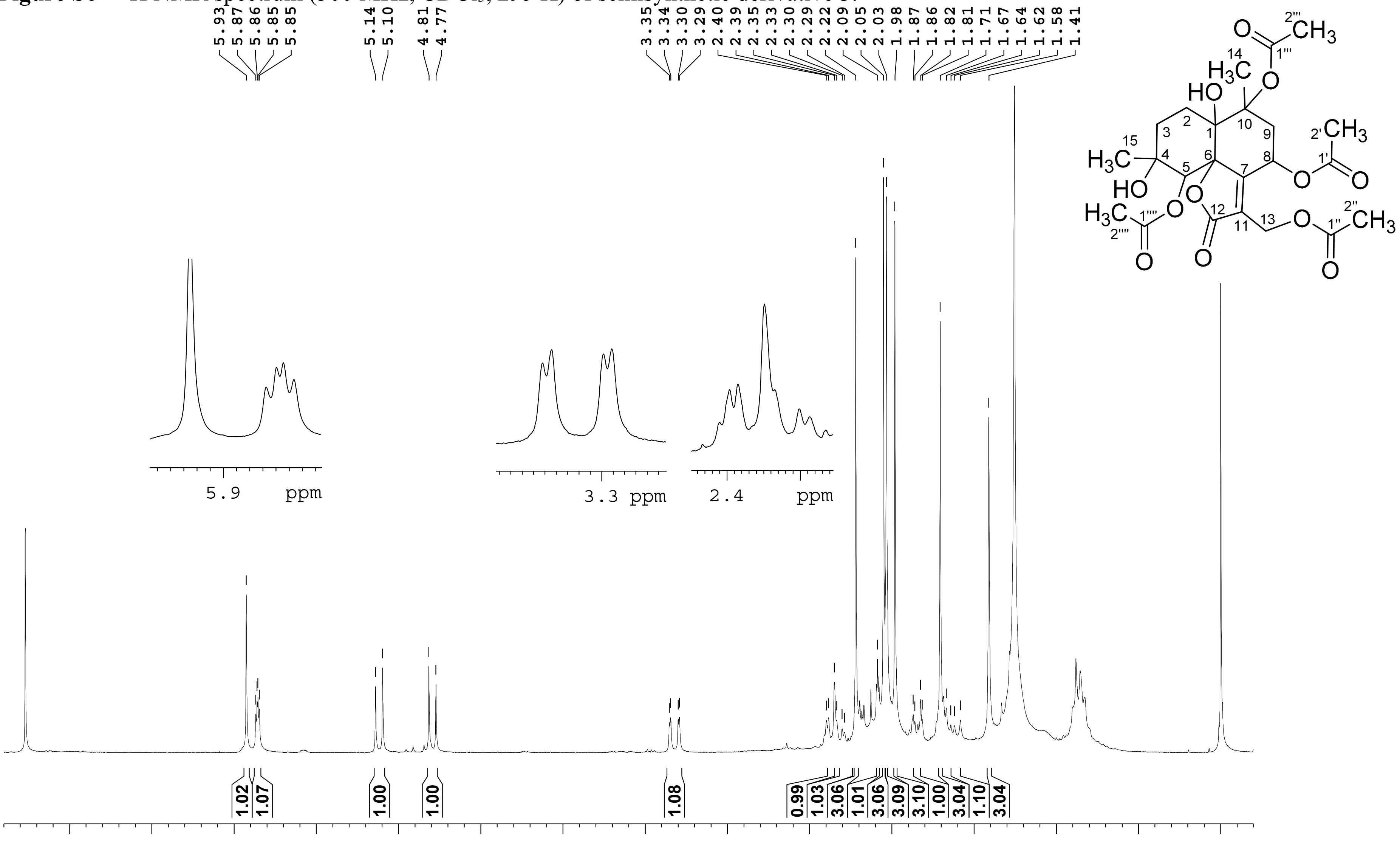

Chemical shift, in ppm (referenced to TMS - δ 0.00).

Figure S7 – HSQC correlation map ($CDCl_3$, 295 K) of semisynthetic derivative **3**.

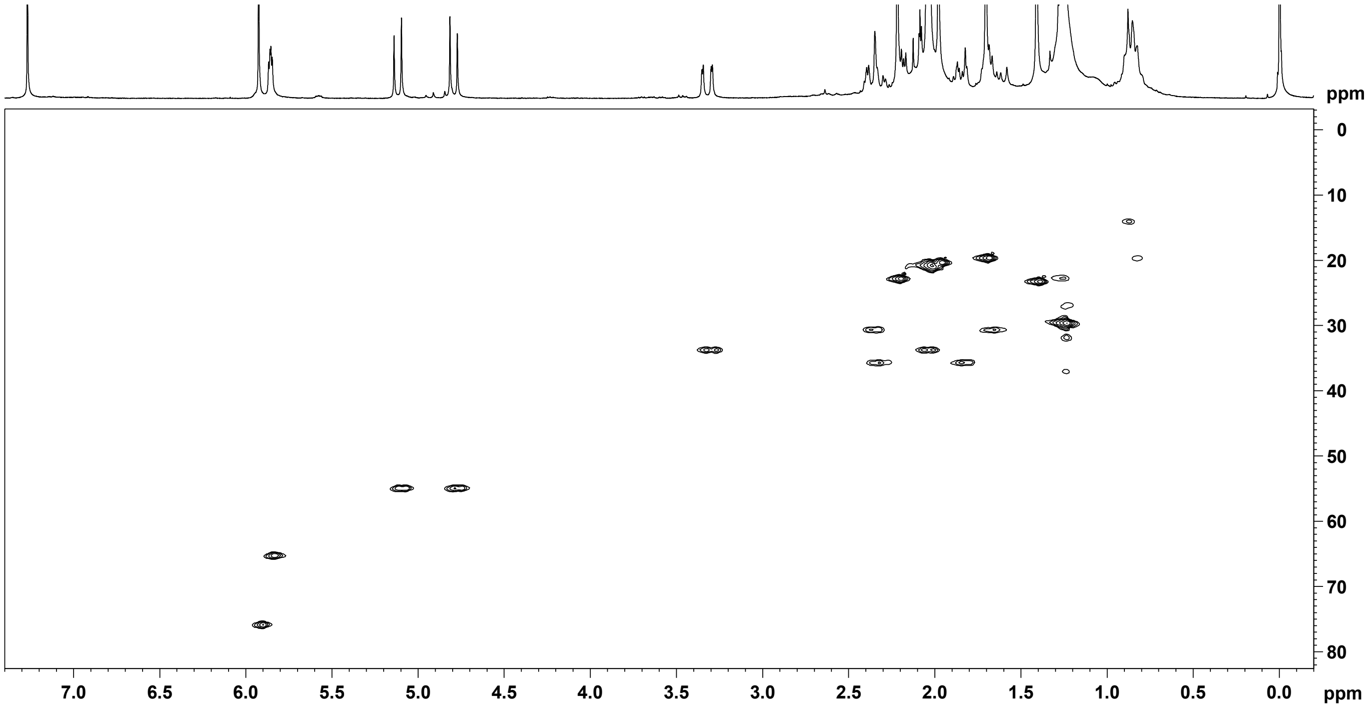

Chemical shift, in ppm (referenced to TMS - δ 0.00).

Figure S8 – HMBC correlation map ($CDCl_3$, 295 K) of semisynthetic derivative **3**.

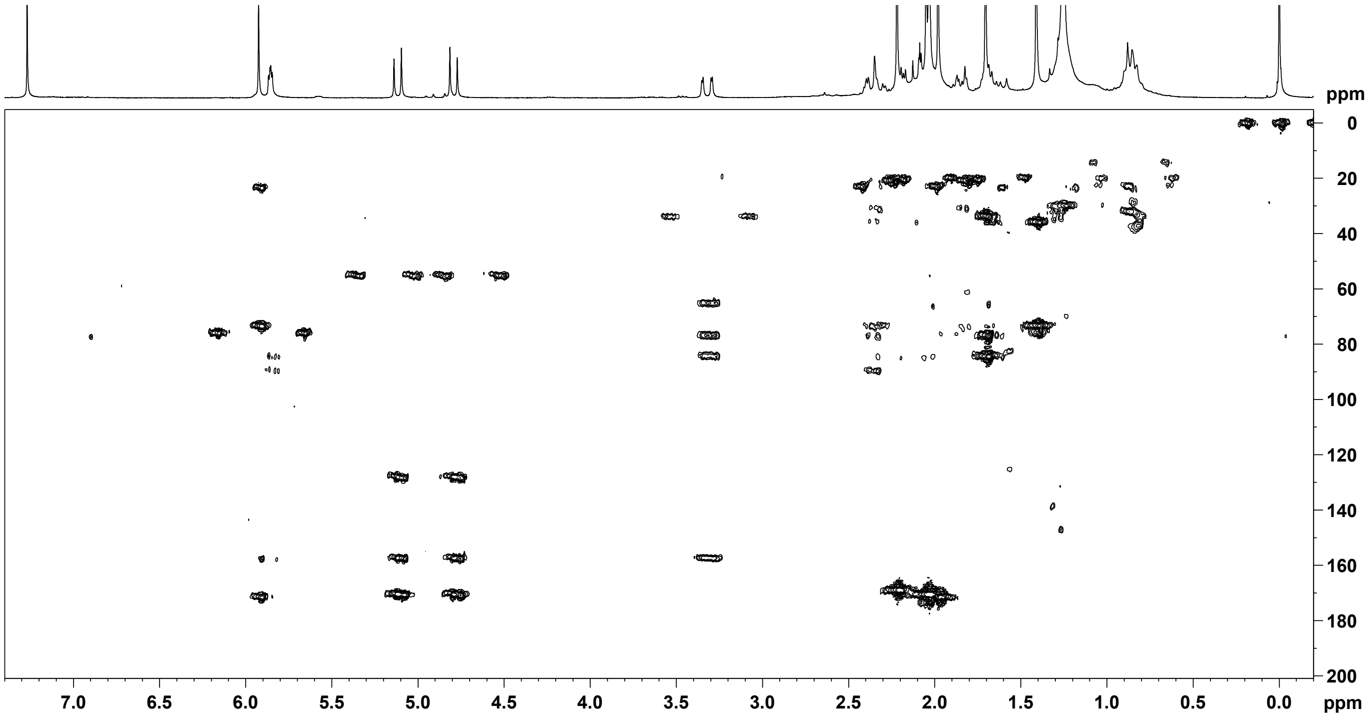

Chemical shift, in ppm (referenced to TMS - δ 0.00).

Figure S9 – HRESIMS *full scan* (100-1200 Da) spectrum of semisynthetic derivative **3**.

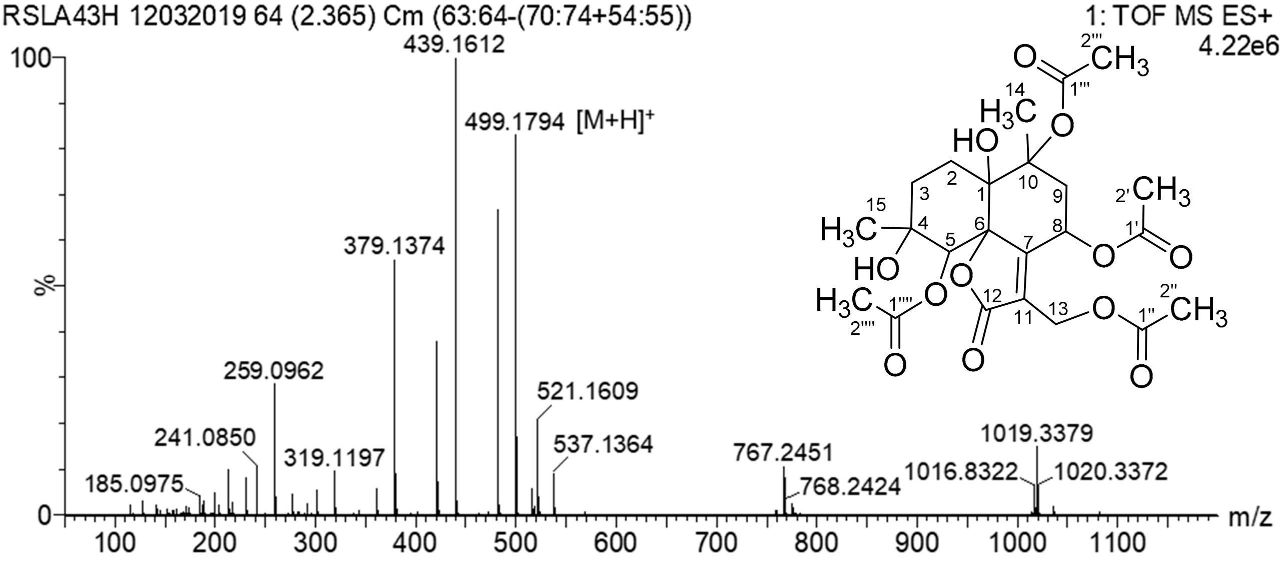

HRESIMS spectrum in positive mode achieved from UPLC-MS analysis.

Figure S10 – UV spectrum (200-400 nm) of semisynthetic derivative **3**.

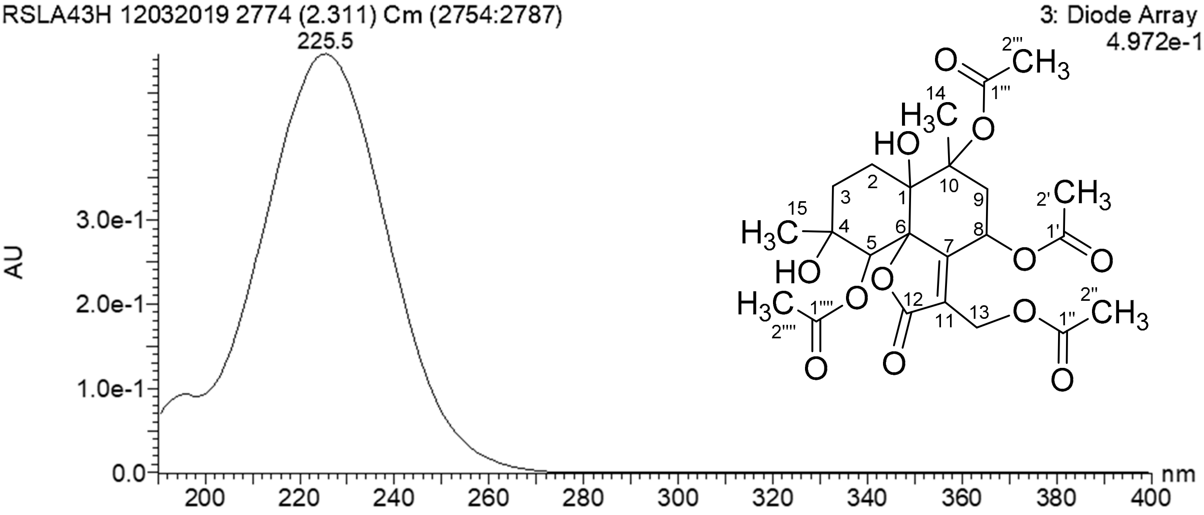

UV spectrum achieved from UPLC-PDA-MS analysis.

Figure S11 – ^{1}H NMR spectrum (300 MHz, $CDCl_3$, 295 K) of semisynthetic derivative **4**.

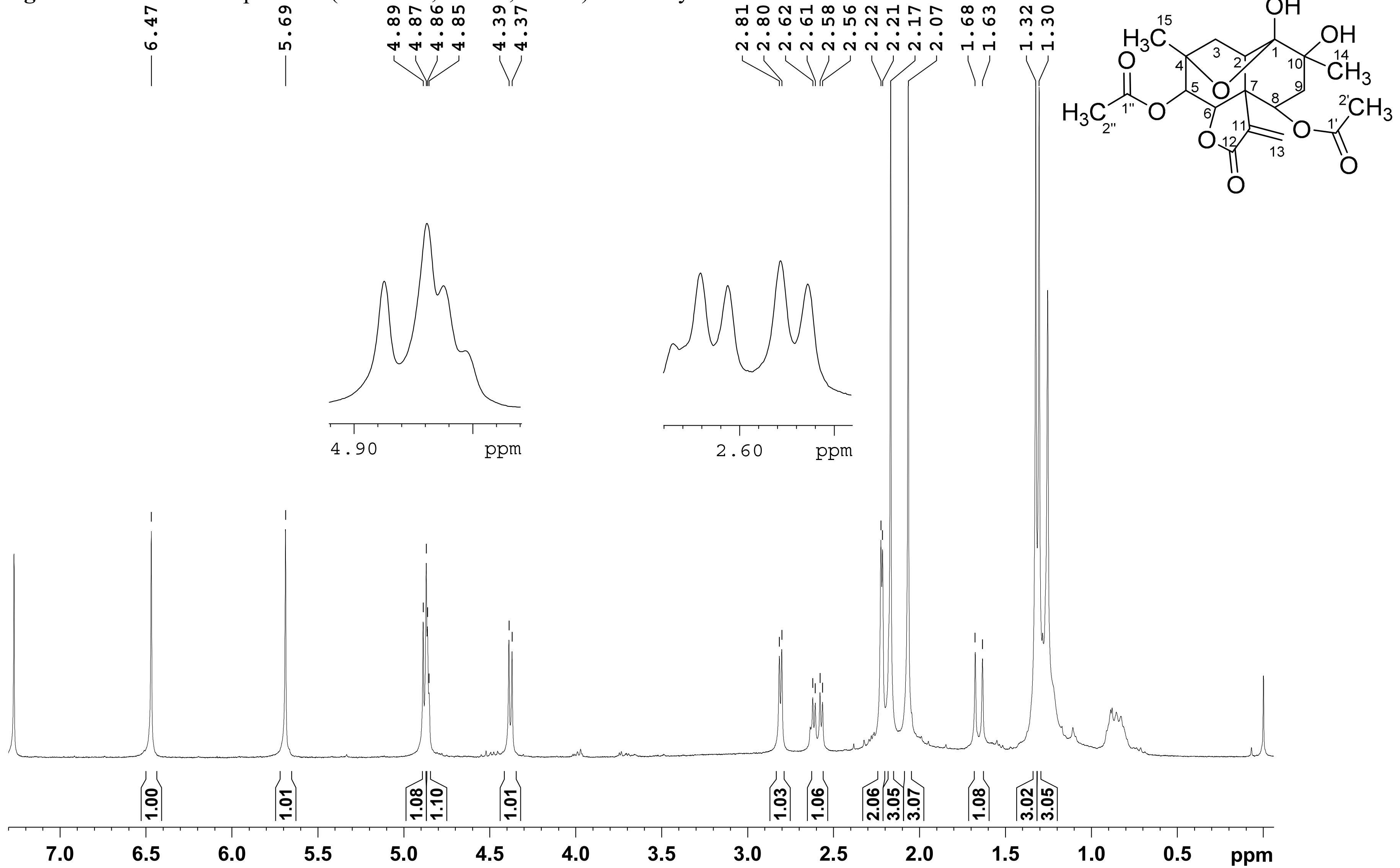

Chemical shift, in ppm (referenced to TMS - δ 0.00).

Figure S12 – HSQC correlation map ($CDCl_3$, 295 K) of semisynthetic derivative **4**.

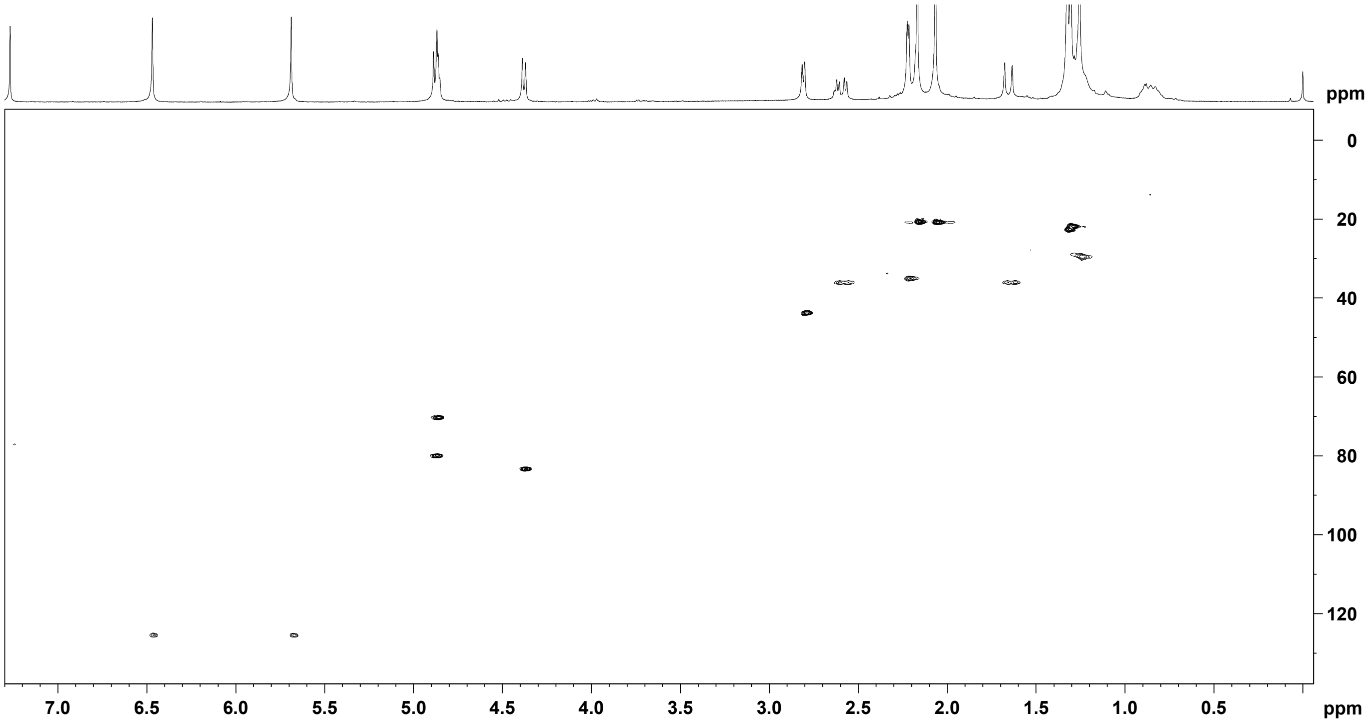

Chemical shift, in ppm (referenced to TMS - δ 0.00).

Figure S13 – HMBC correlation map ($CDCl_3$, 295 K) of semisynthetic derivative **4**.

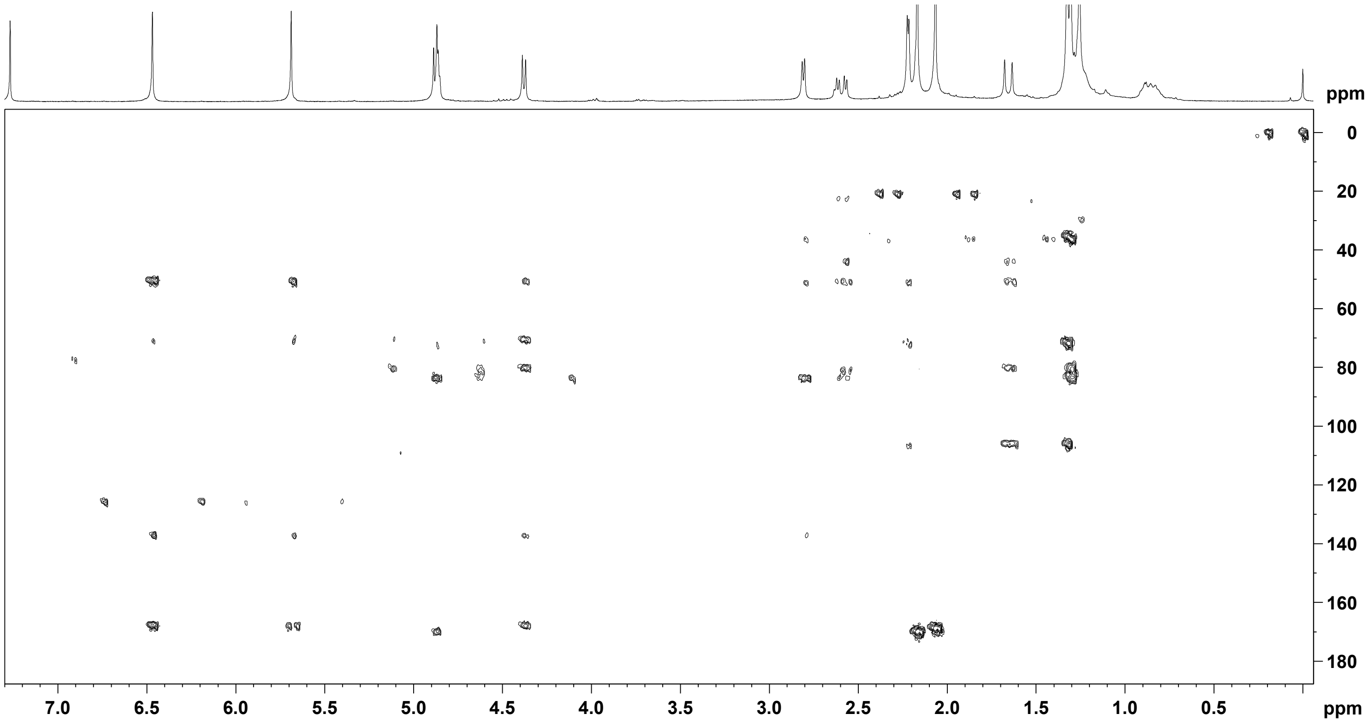

Chemical shift, in ppm (referenced to TMS - δ 0.00).

Figure S14 – HRESIMS *full scan* (100-1200 Da) spectrum of semisynthetic derivative **4**.

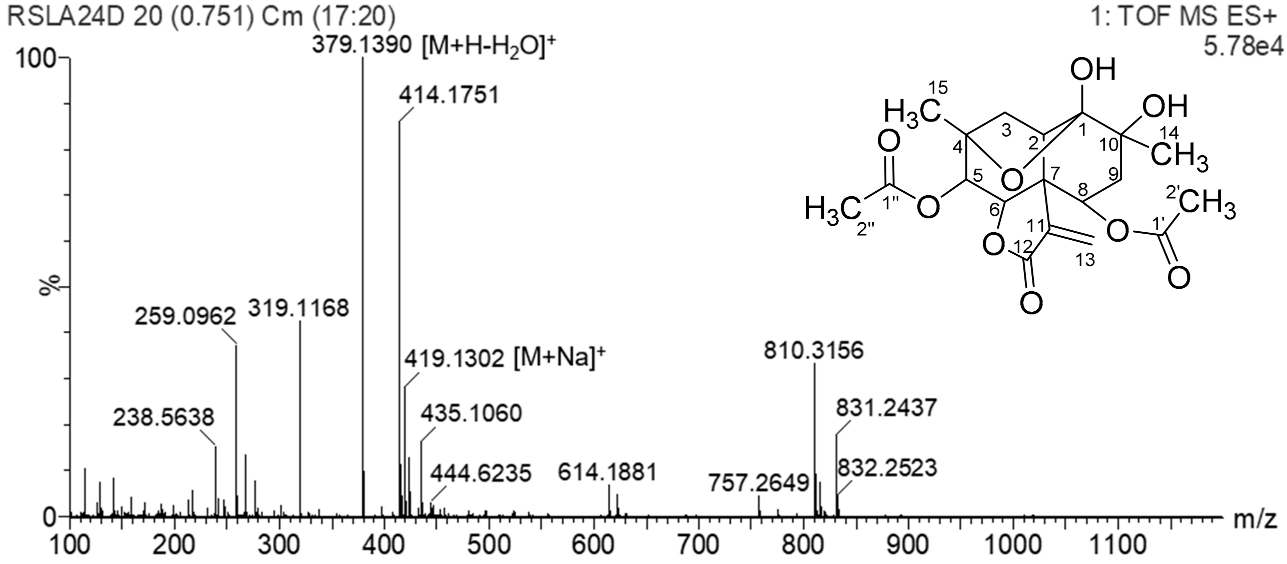

HRESIMS spectrum in positive mode achieved from UPLC-MS analysis.

Figure S15 – UV spectrum (200-400 nm) of semisynthetic derivative **4**.

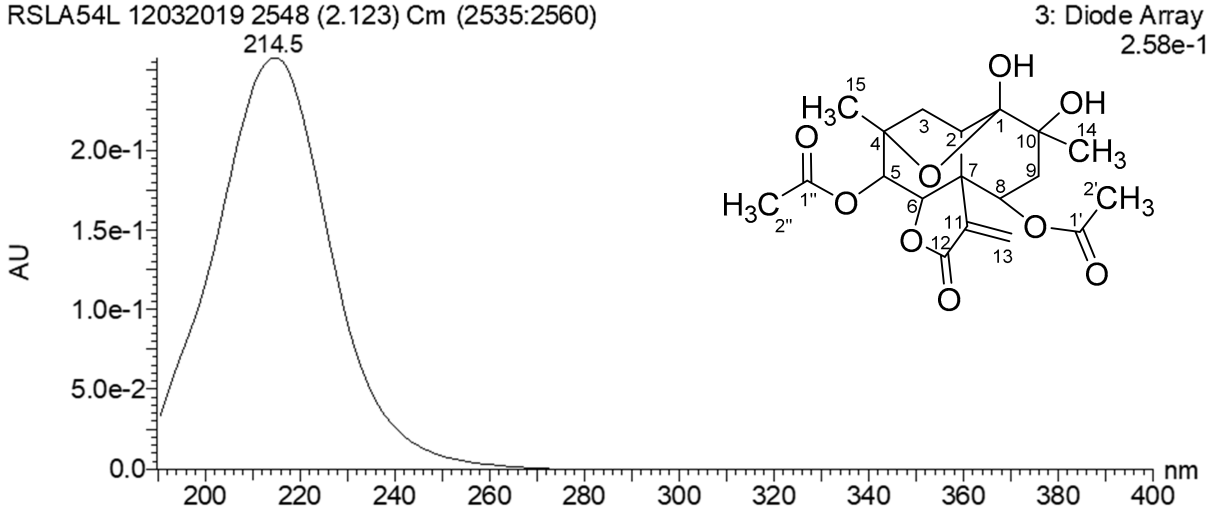

UV spectrum achieved from UPLC-PDA-MS analysis.

Figure S16 – ^{1}H NMR spectrum (300 MHz, $CDCl_3$, 295 K) of semisynthetic derivative **5**.

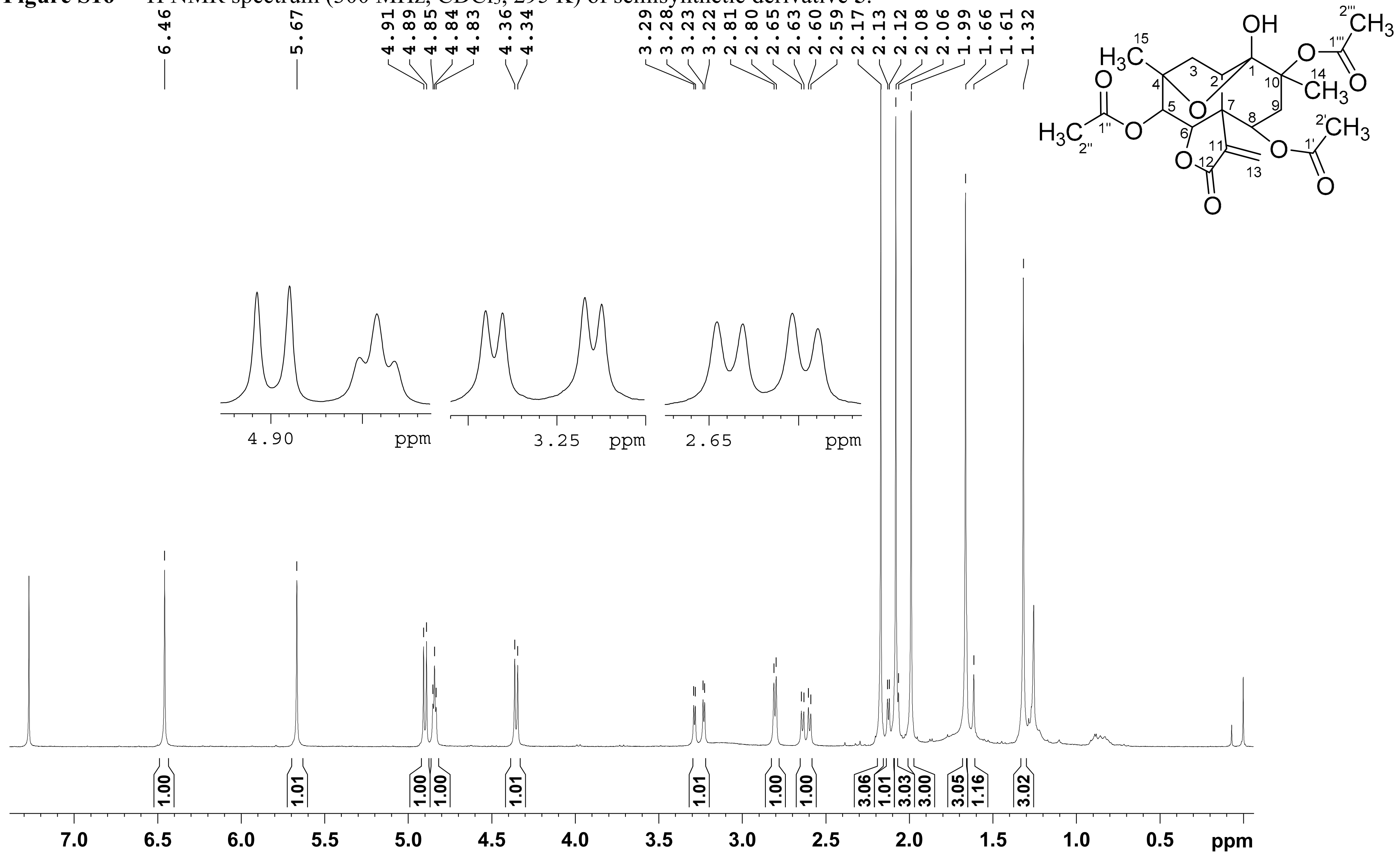

Chemical shift, in ppm (referenced to TMS - δ 0.00).

Figure S17 – HSQC correlation map ($CDCl_3$, 295 K) of semisynthetic derivative **5**.

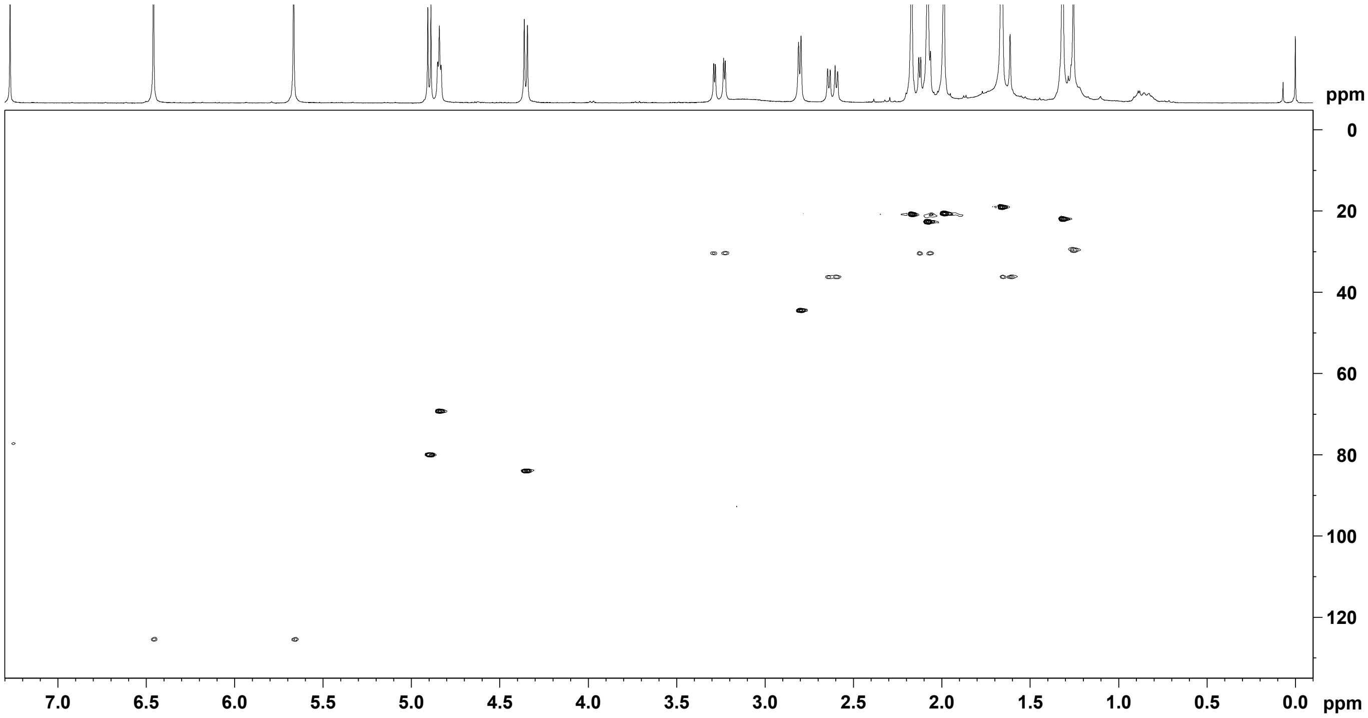

Chemical shift, in ppm (referenced to TMS - δ 0.00).

Figure S18 – HMBC correlation map ($CDCl_3$, 295 K) of semisynthetic derivative **5**.

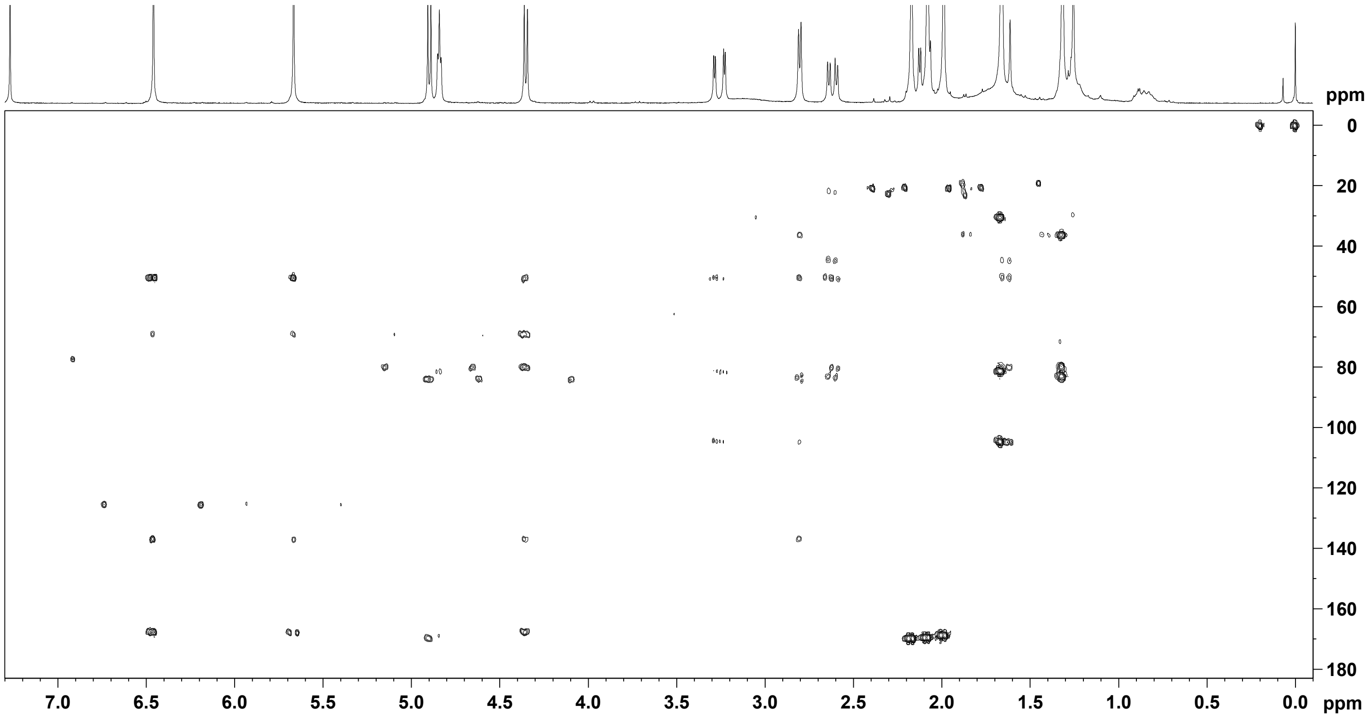

Chemical shift, in ppm (referenced to TMS - δ 0.00).

Figure S19 – HRESIMS *full scan* (100-1200 Da) spectrum of semisynthetic derivative **5**.

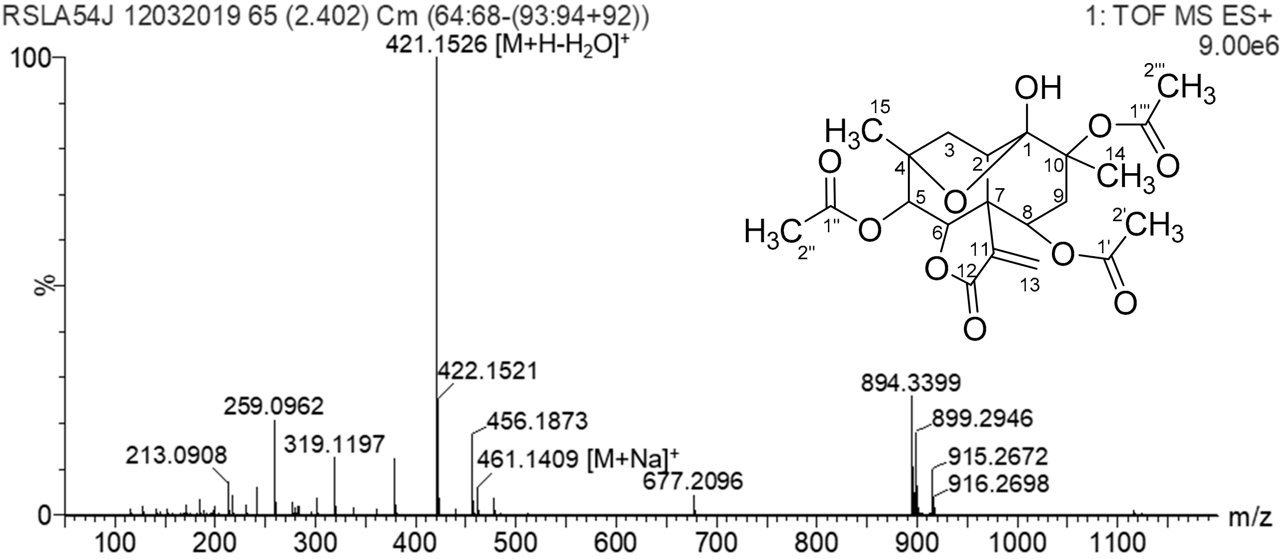

HRESIMS spectrum in positive mode achieved from UPLC-MS analysis.

Figure S20 – UV spectrum (200-400 nm) of semisynthetic derivative **5**.

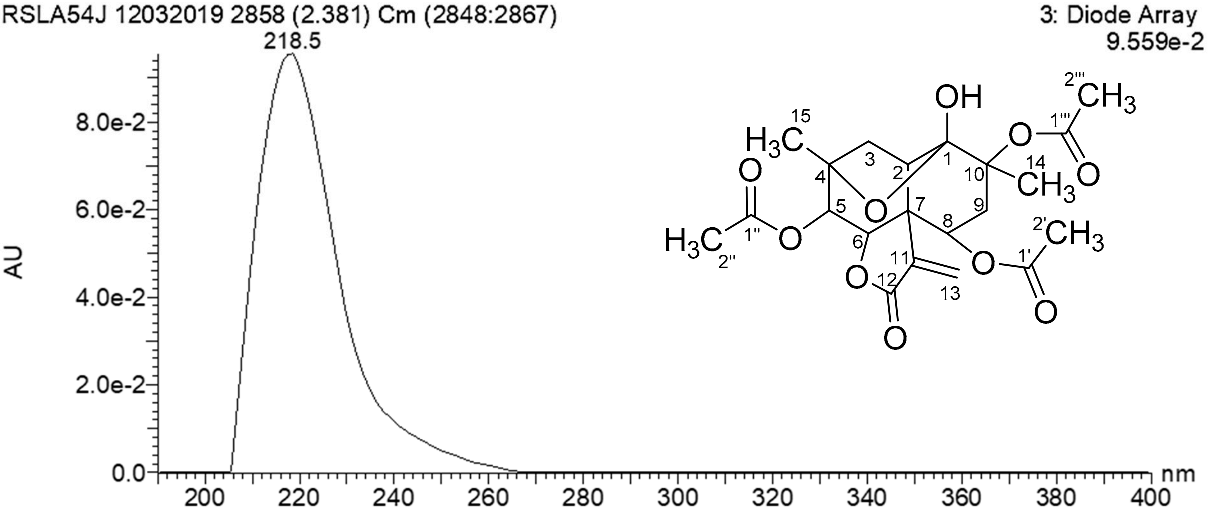

UV spectrum achieved from UPLC-PDA-MS analysis.

Figure S21 – ^{1}H NMR spectrum (300 MHz, $CDCl_3$, 295 K) of semisynthetic derivative **6**.

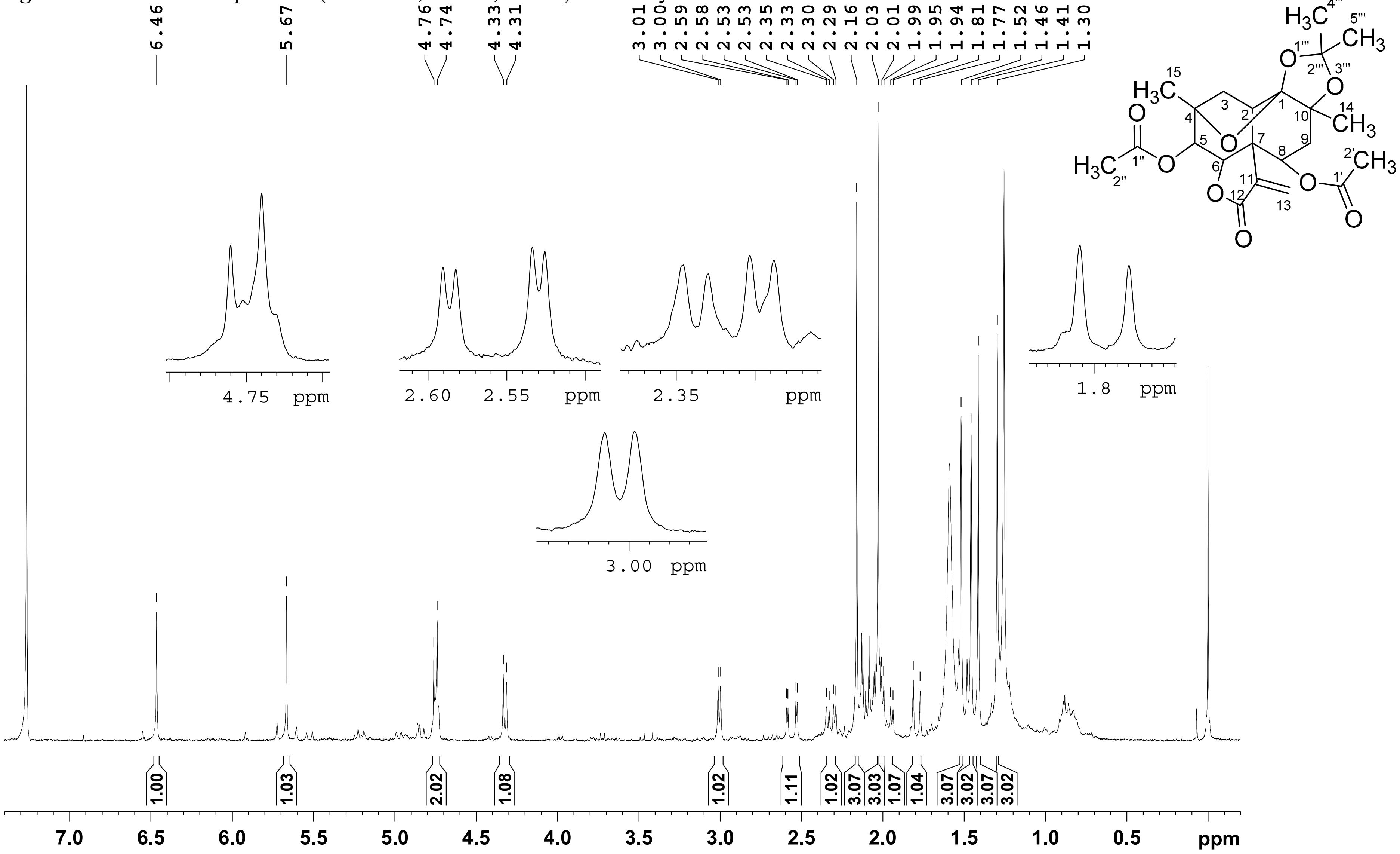

Chemical shift, in ppm (referenced to TMS - δ 0.00).

Figure S22 – HSQC correlation map ($CDCl_3$, 295 K) of semisynthetic derivative **6**.

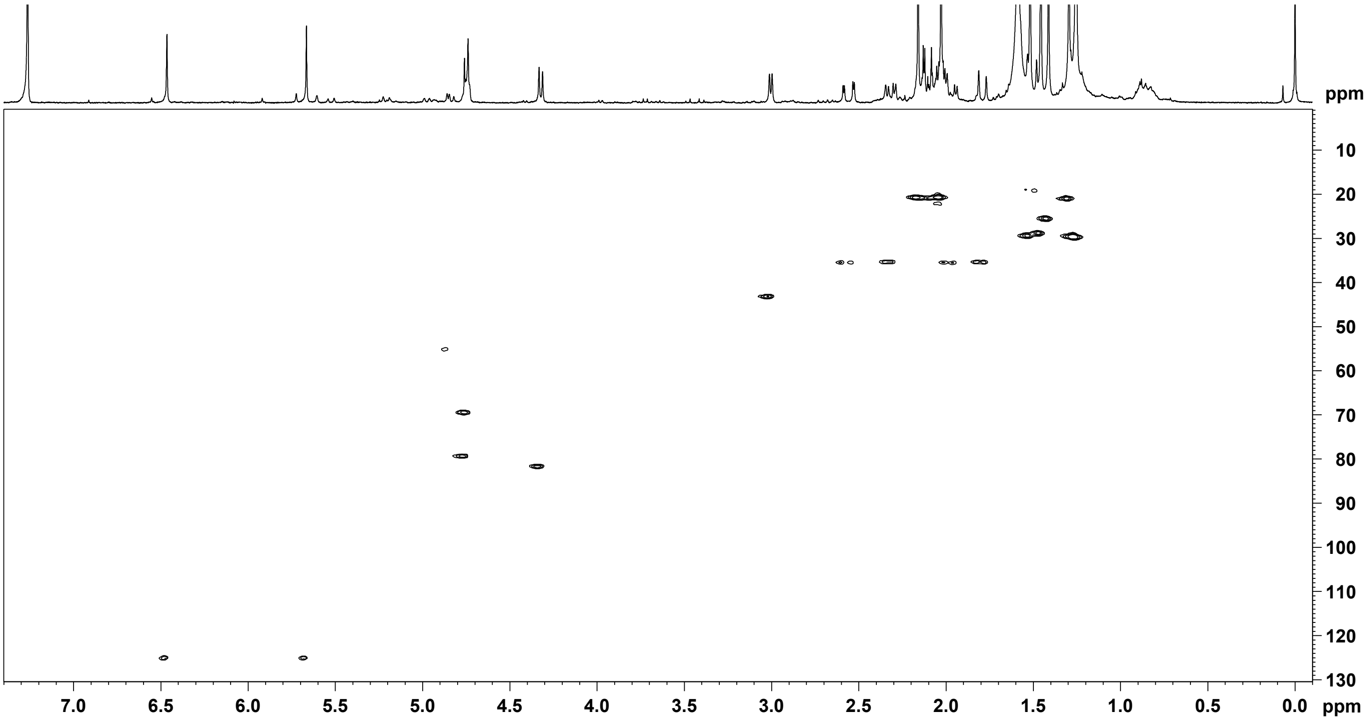

Chemical shift, in ppm (referenced to TMS - δ 0.00).

Figure S23 – HMBC correlation map ($CDCl_3$, 295 K) of semisynthetic derivative **6**.

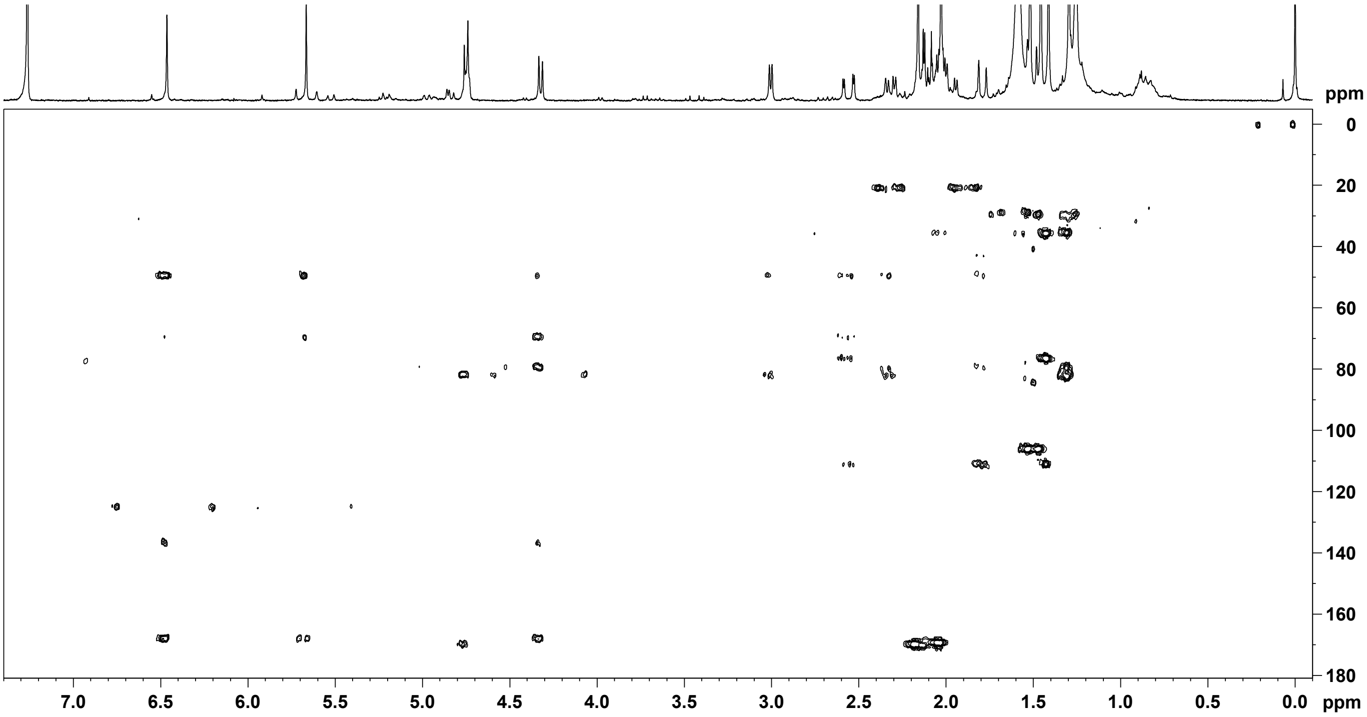

Chemical shift, in ppm (referenced to TMS - δ 0.00).

Figure S24 – HRESIMS *full scan* (100-1200 Da) spectrum of semisynthetic derivative **6**.

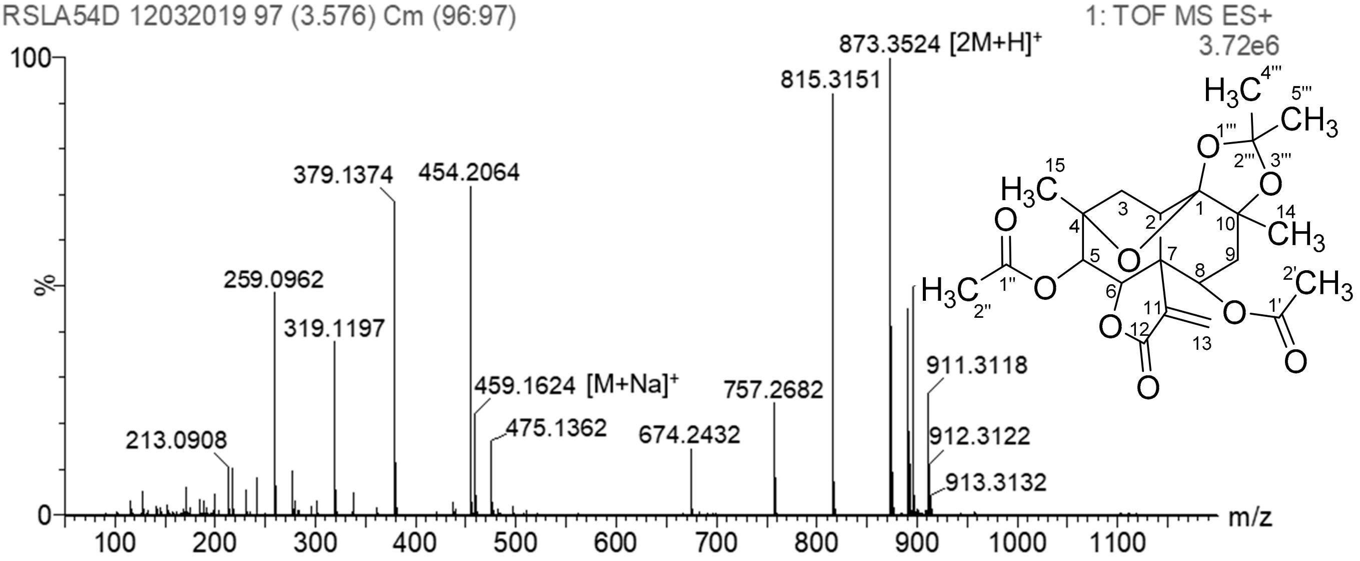

HRESIMS spectrum in positive mode achieved from UPLC-MS analysis.

Figure S25 – UV spectrum (200-400 nm) of semisynthetic derivative **6**.

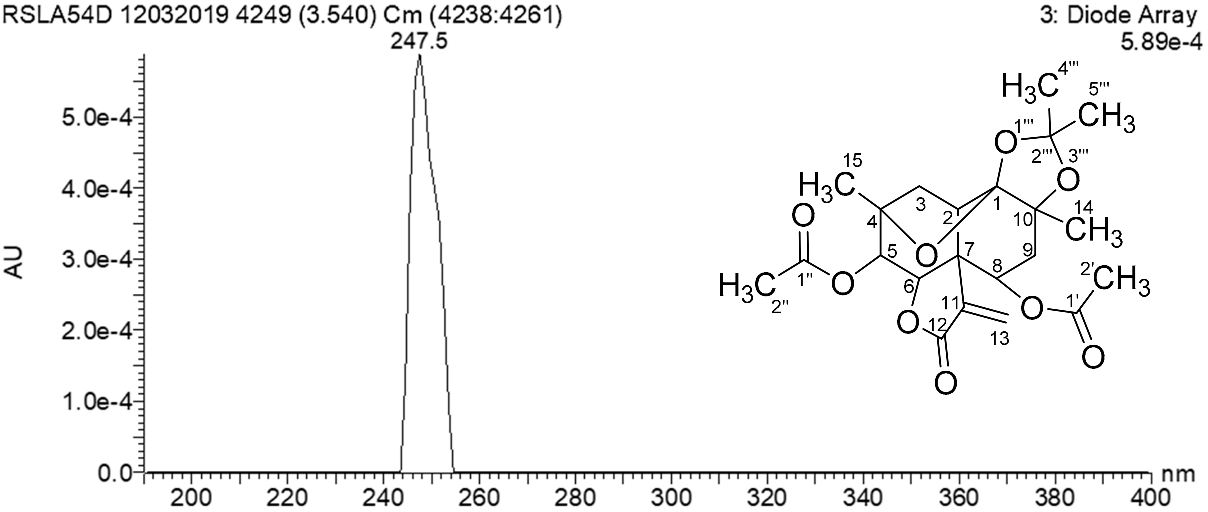

UV spectrum achieved from UPLC-PDA-MS analysis.

Figure S26 – ^{1}H NMR spectrum (300 MHz, $CDCl_3$, 295 K) of semisynthetic derivative **7**.

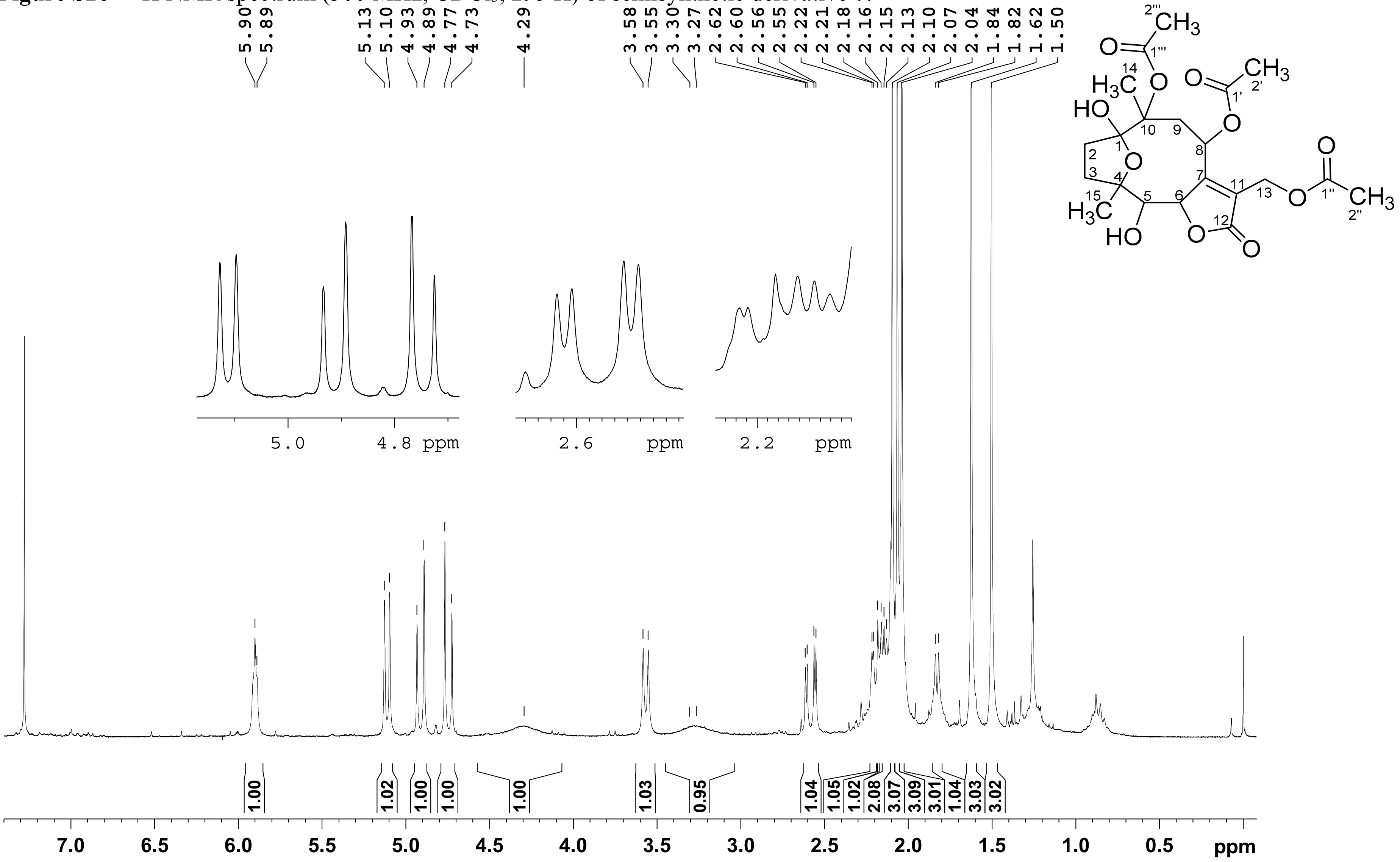

Chemical shift, in ppm (referenced to TMS - δ 0.00).

Figure S27 – HSQC correlation map ($CDCl_3$, 295 K) of semisynthetic derivative **7**.

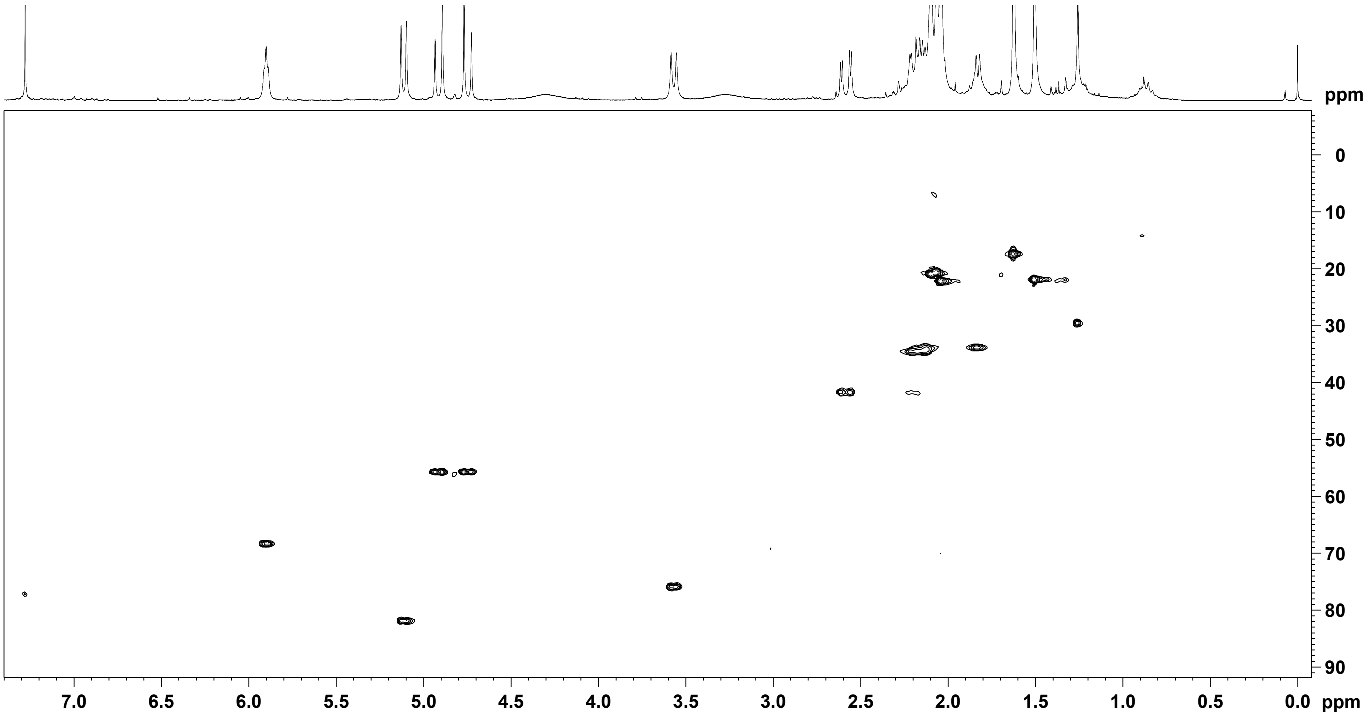

Chemical shift, in ppm (referenced to TMS - δ 0.00).

Figure S28 – HMBC correlation map ($CDCl_3$, 295 K) of semisynthetic derivative **7**.

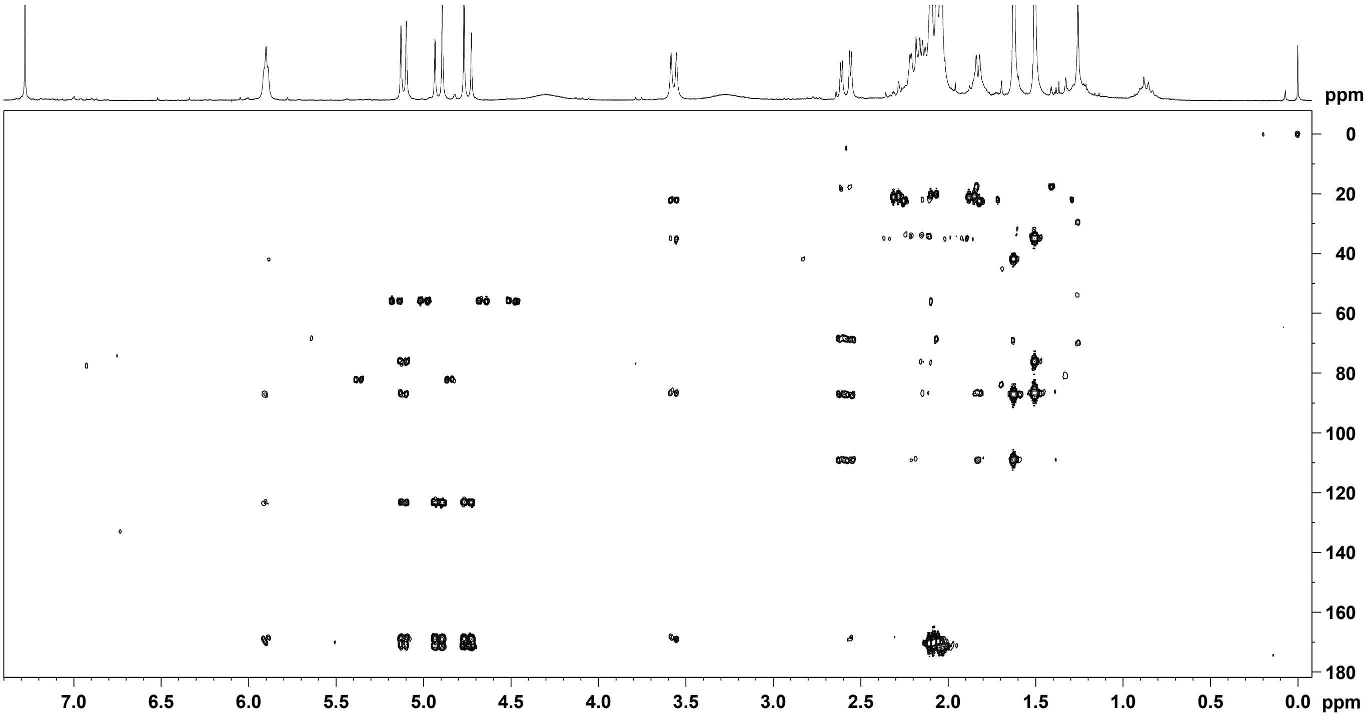

Chemical shift, in ppm (referenced to TMS - δ 0.00).

Figure S29 – HRESIMS *full scan* (100-1200 Da) spectrum of semisynthetic derivative **7**.

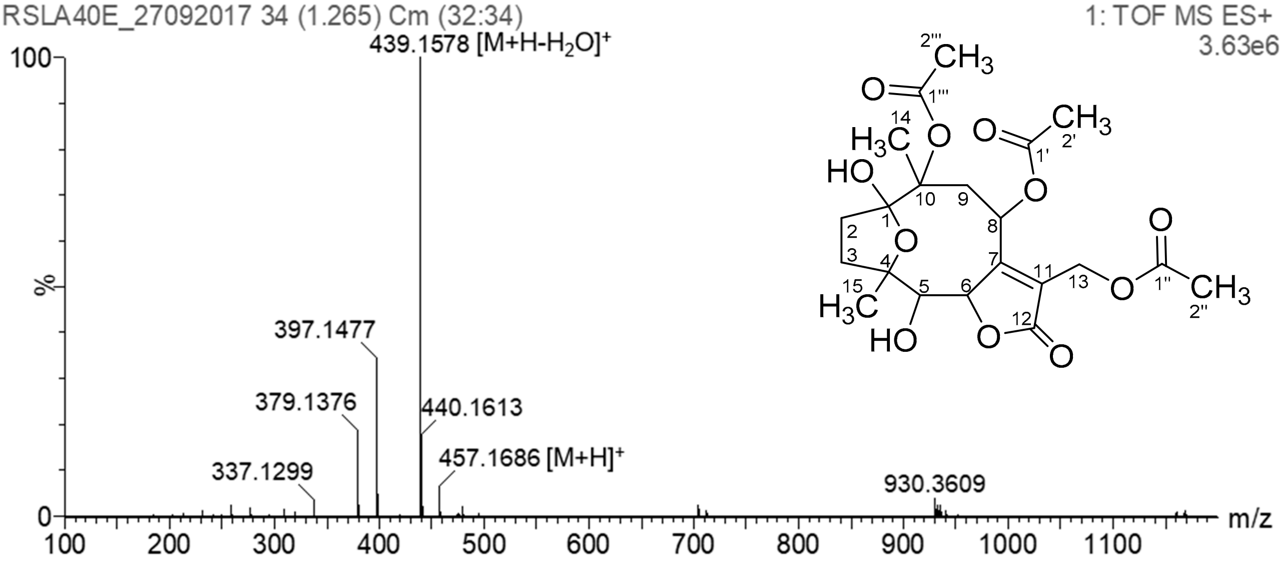

HRESIMS spectrum in positive mode achieved from UPLC-MS analysis.

Figure S30 – ^{1}H NMR spectrum (300 MHz, CDCl3, 295 K) of semisynthetic derivative **8**.

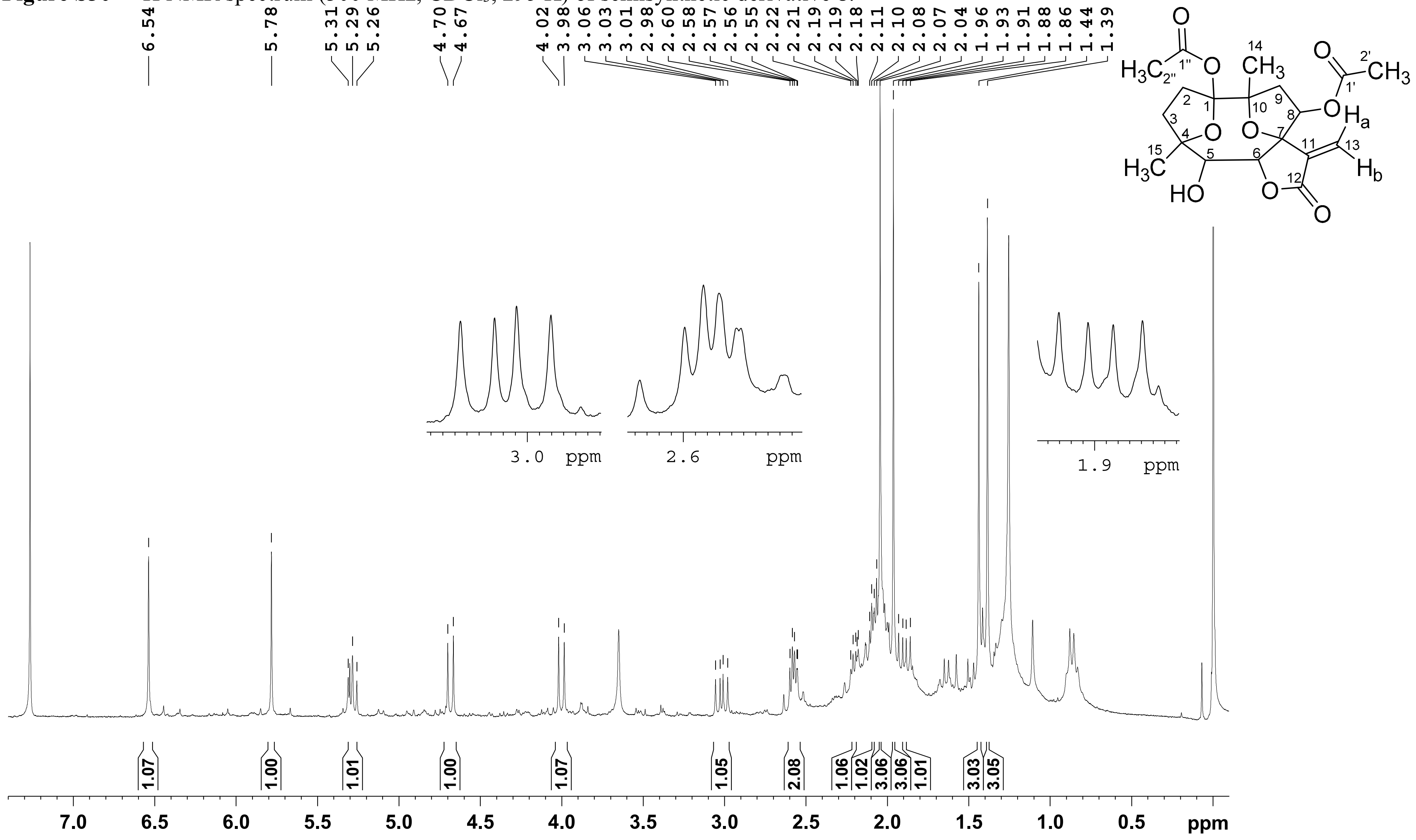

Chemical shift, in ppm (referenced to TMS - δ 0.00).

Figure S31 – HSQC correlation map ($CDCl_3$, 295 K) of semisynthetic derivative **8**.

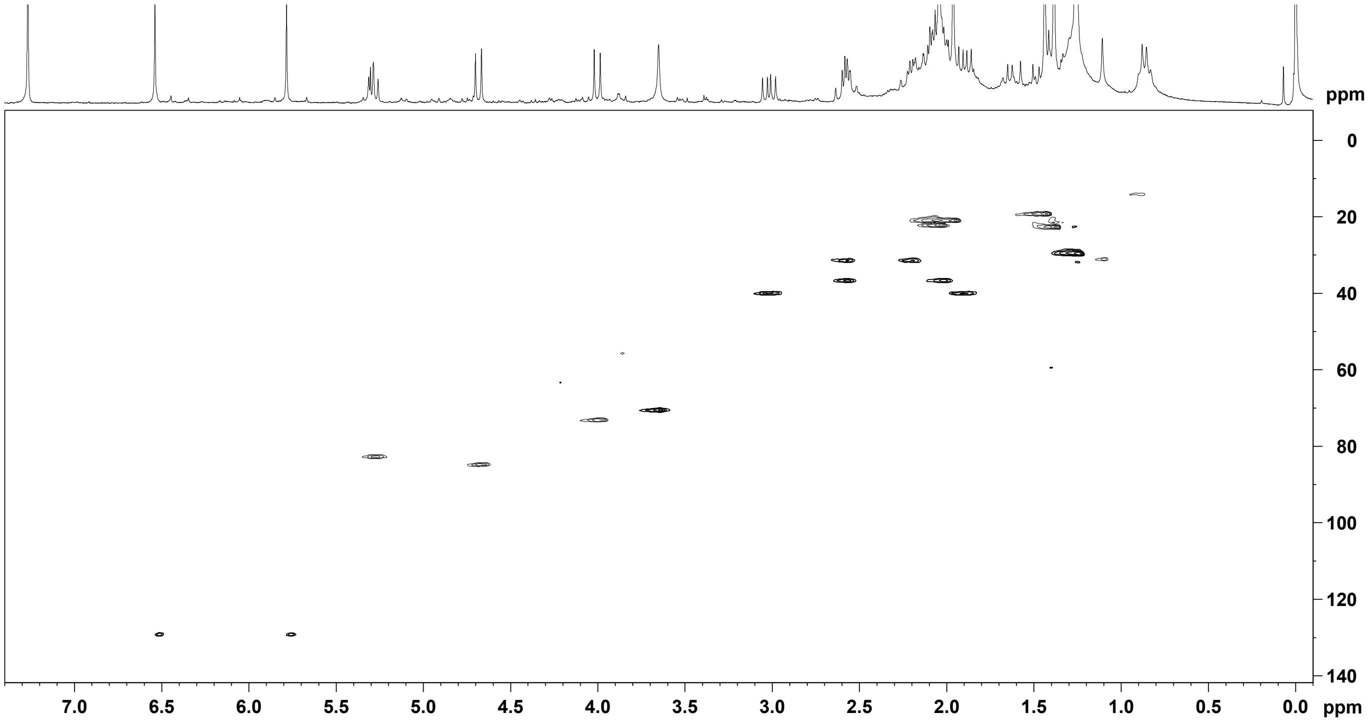

Chemical shift, in ppm (referenced to TMS - δ 0.00).

Figure S32 – HMBC correlation map ($CDCl_3$, 295 K) of semisynthetic derivative **8**.

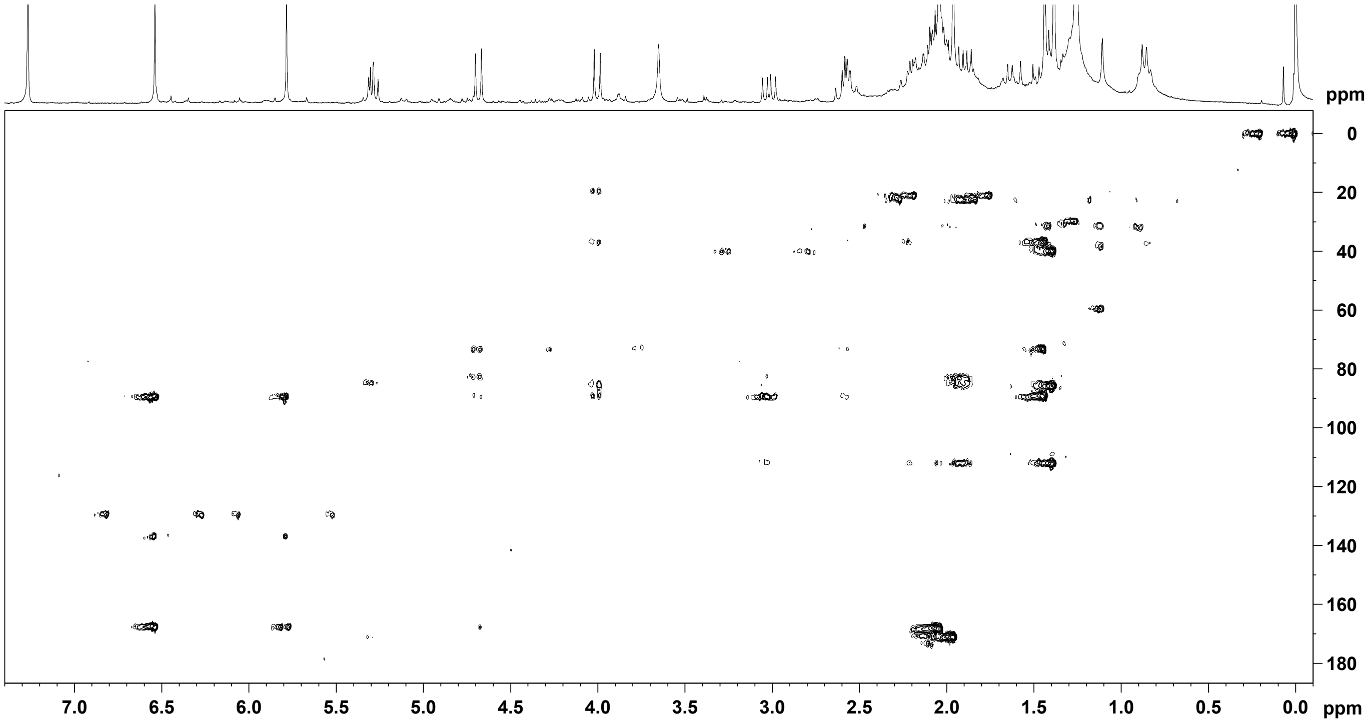

Chemical shift, in ppm (referenced to TMS - δ 0.00).

Figure S33 – HRESIMS *full scan* (100-1200 Da) spectrum of semisynthetic derivative **8**.

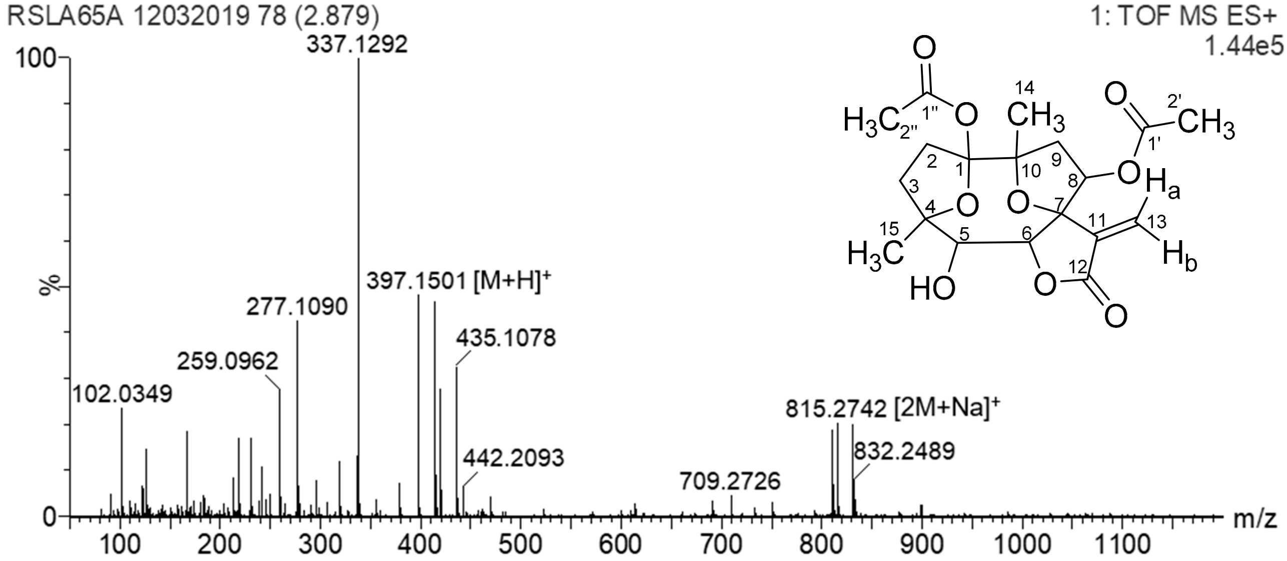

HRESIMS spectrum in positive mode achieved from UPLC-MS analysis.

Figure S34 – UV spectrum (200-400 nm) of semisynthetic derivative **8**.

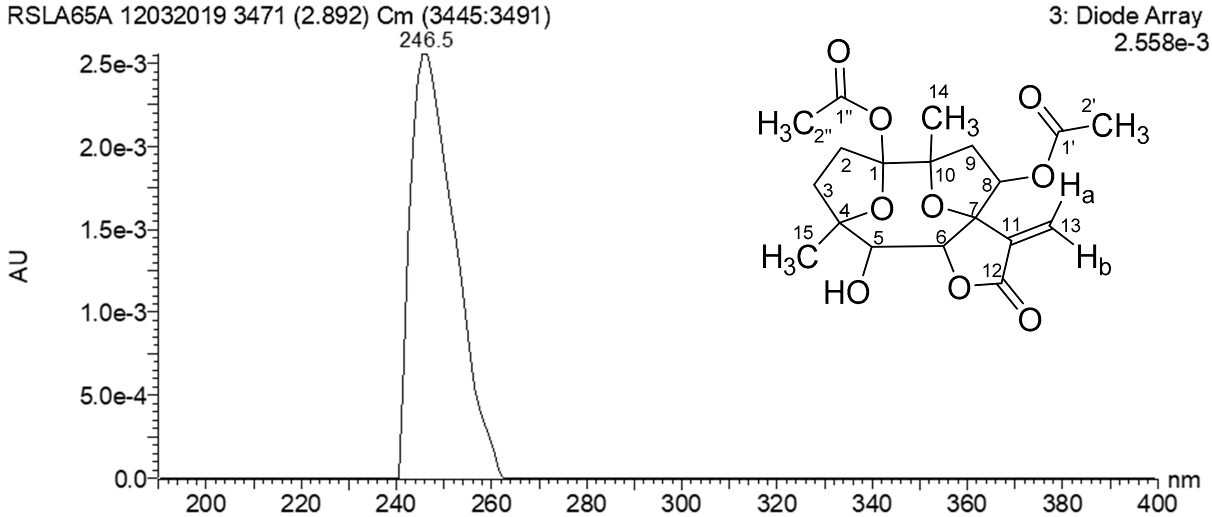

UV spectrum achieved from UPLC-PDA-MS analysis.

Figure S35 – ^{1}H NMR spectrum (300 MHz, $CDCl_3$, 295 K) of semisynthetic derivative **9**.

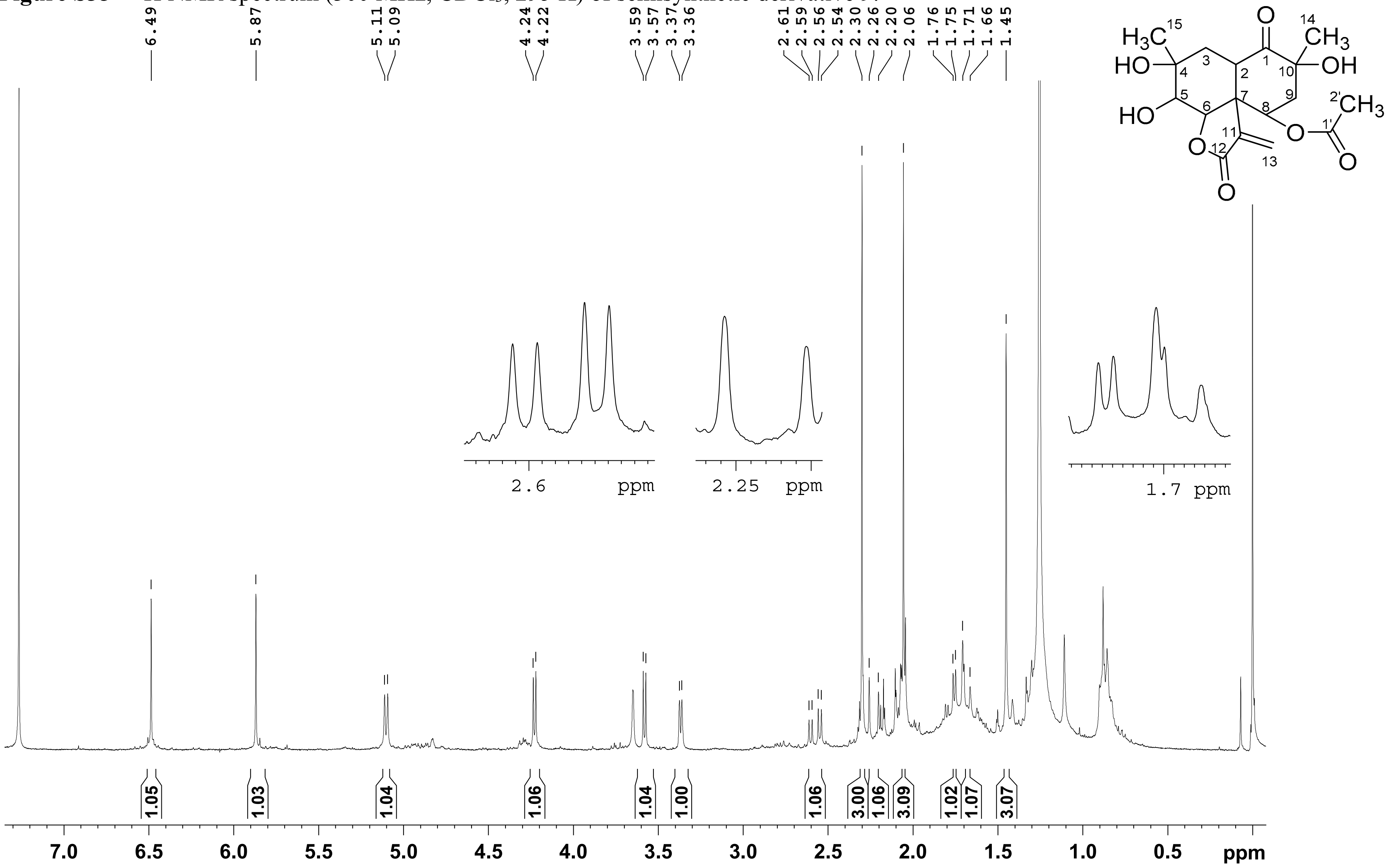

Chemical shift, in ppm (referenced to TMS - δ 0.00).

Figure S36 – HSQC correlation map (CDCl3, 295 K) of semisynthetic derivative **9**.

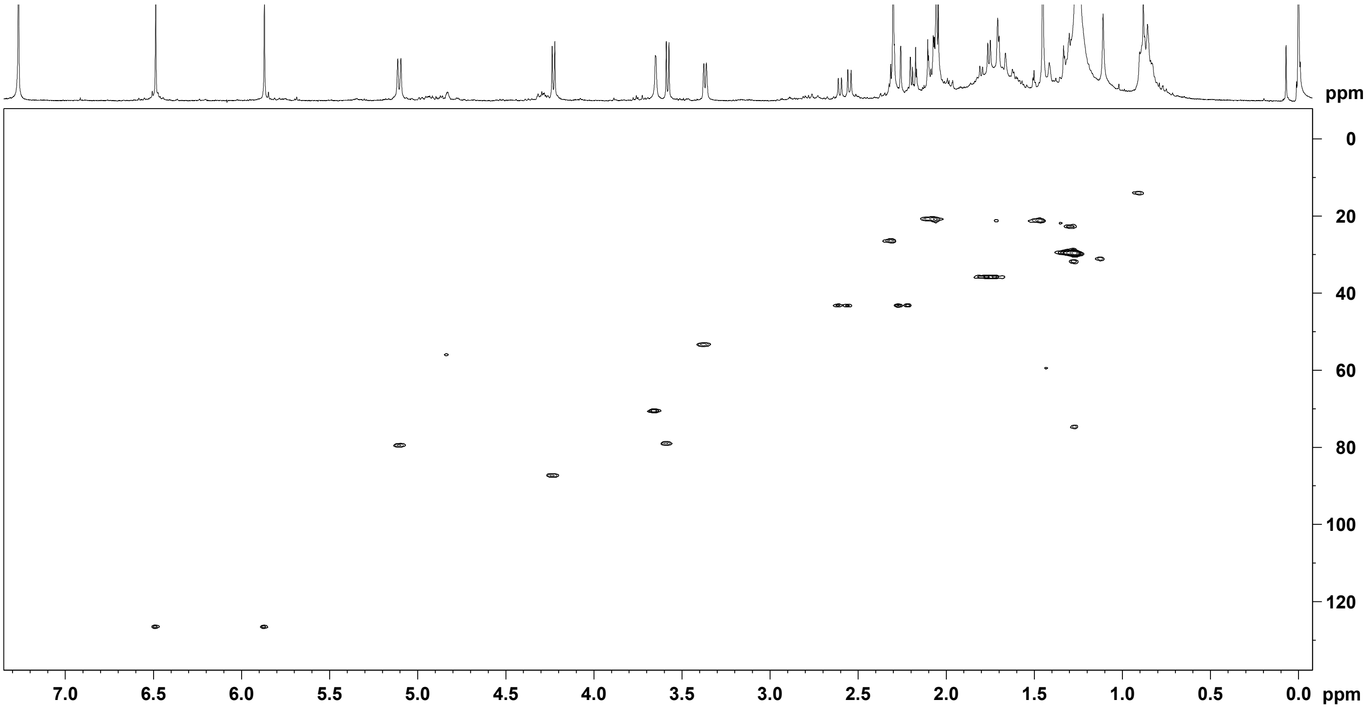

Chemical shift, in ppm (referenced to TMS - δ 0.00).

Figure S37 – HMBC correlation map (CDCl3, 295 K) of semisynthetic derivative **9**.

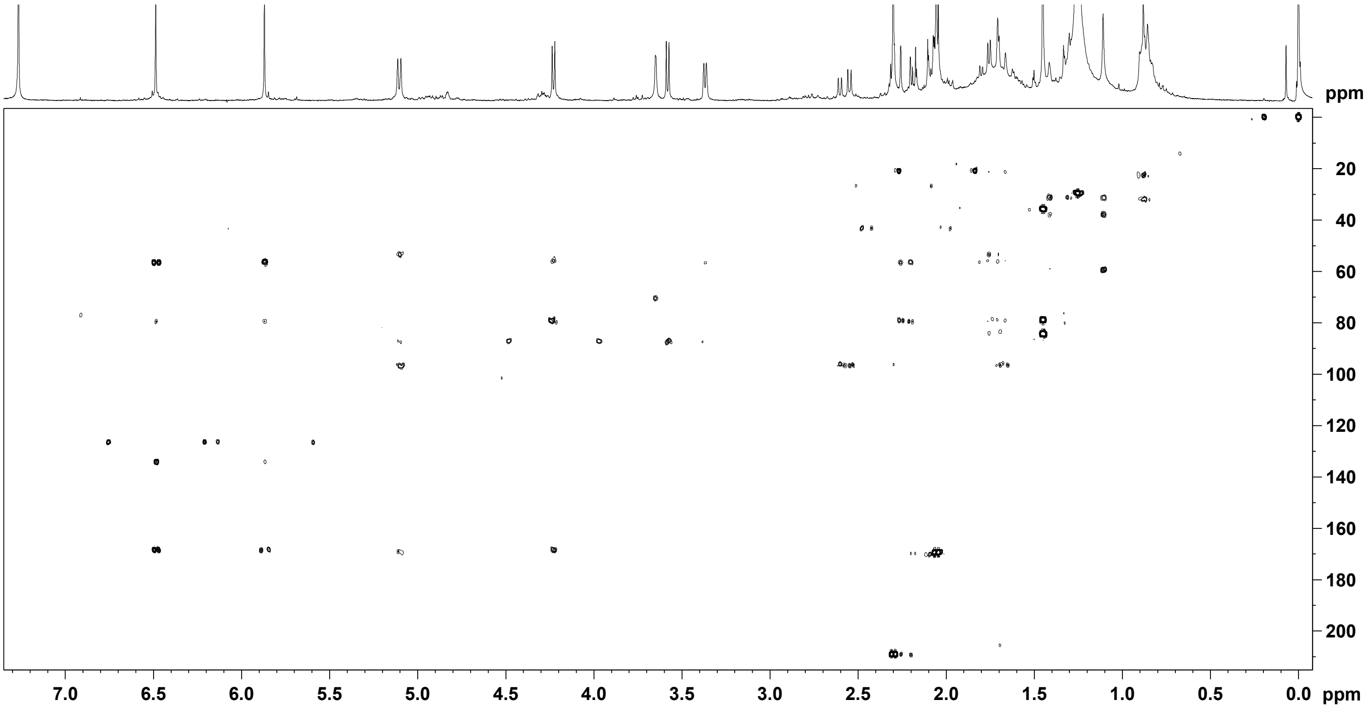

Chemical shift, in ppm (referenced to TMS - δ 0.00).

Figure S38 – HRESIMS *full scan* (100-1200 Da) spectrum of semisynthetic derivative **9**.

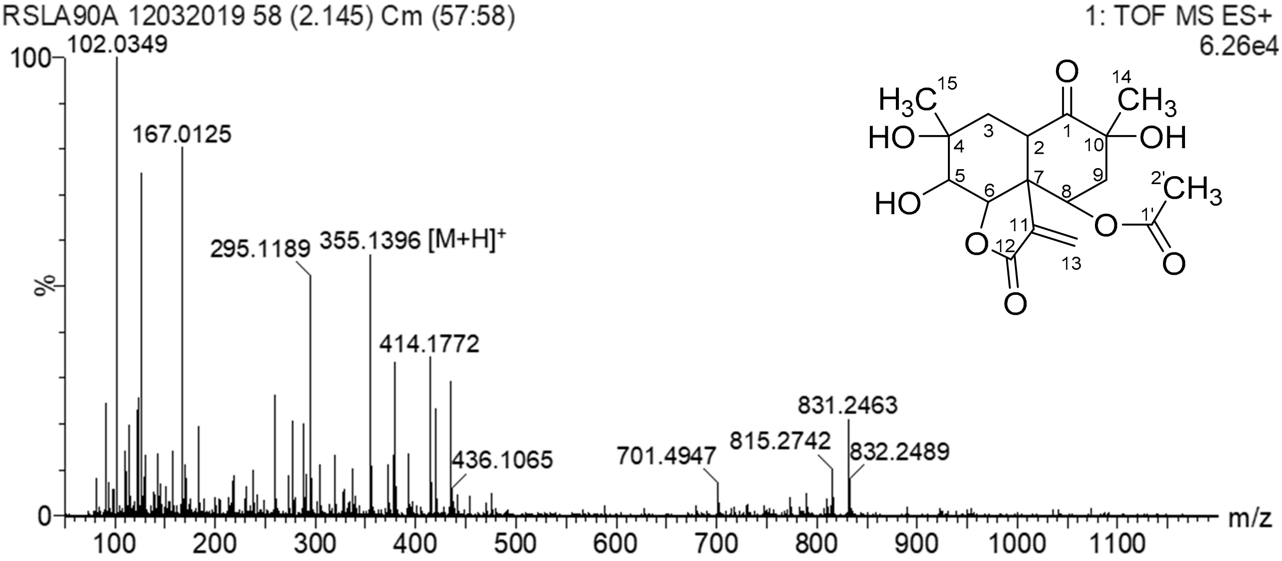

HRESIMS spectrum in positive mode achieved from UPLC-MS analysis.

Figure S39 – UV spectrum (200-400 nm) of semisynthetic derivative **9**.

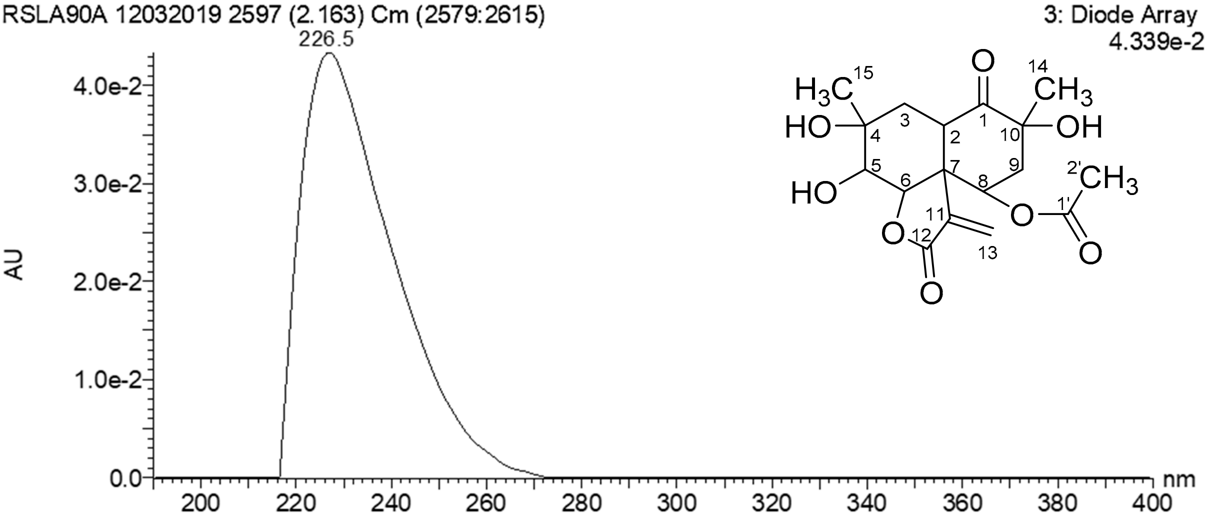

UV spectrum achieved from UPLC-PDA-MS analysis.

Figure S40 – ^{1}H NMR spectrum (300 MHz, $CDCl_3$, 308 K) of semisynthetic derivative **7a**.

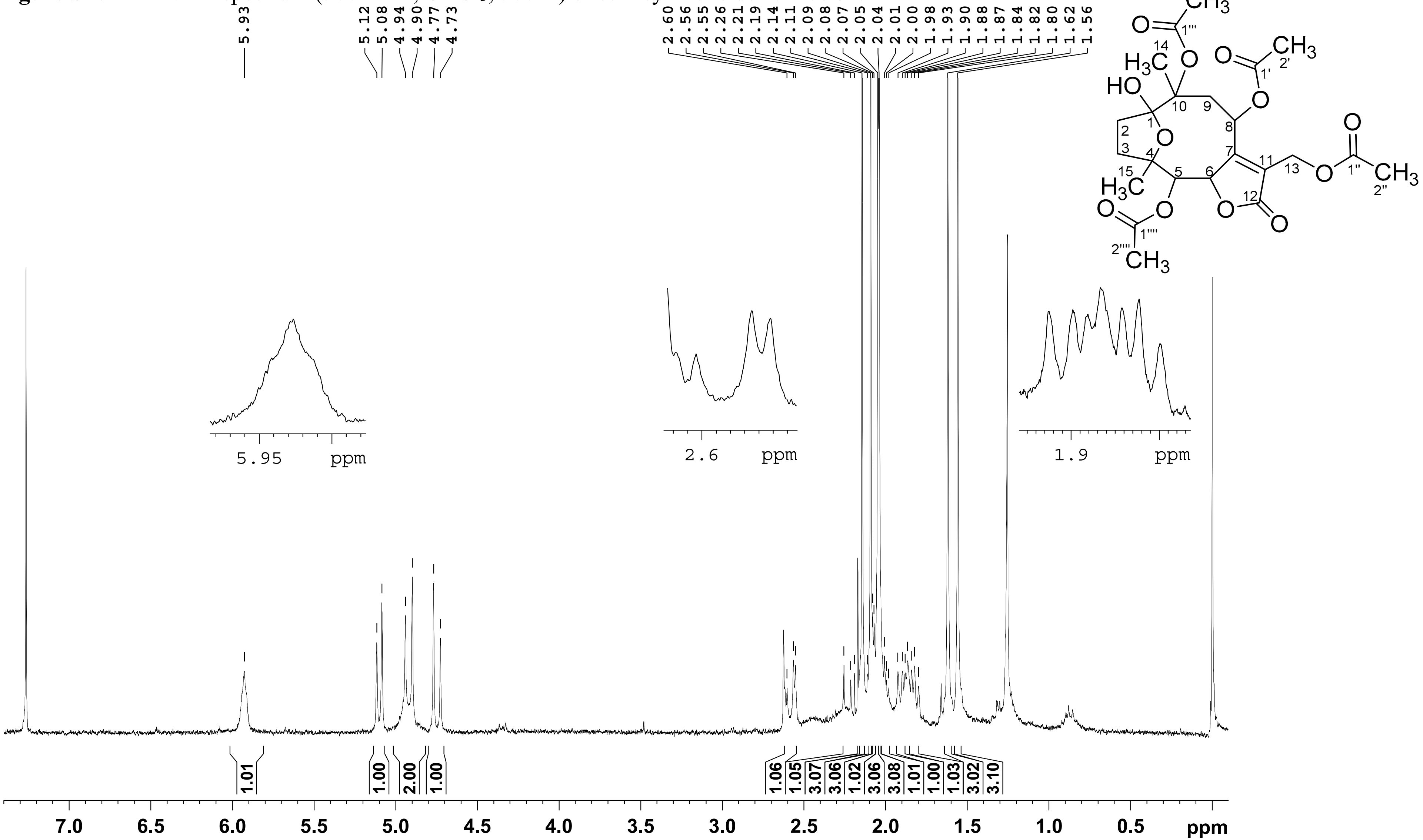

Chemical shift, in ppm (referenced to TMS - δ 0.00).

Figure S41 – HSQC correlation map ($CDCl_3$, 308 K) of semisynthetic derivative **7a**.

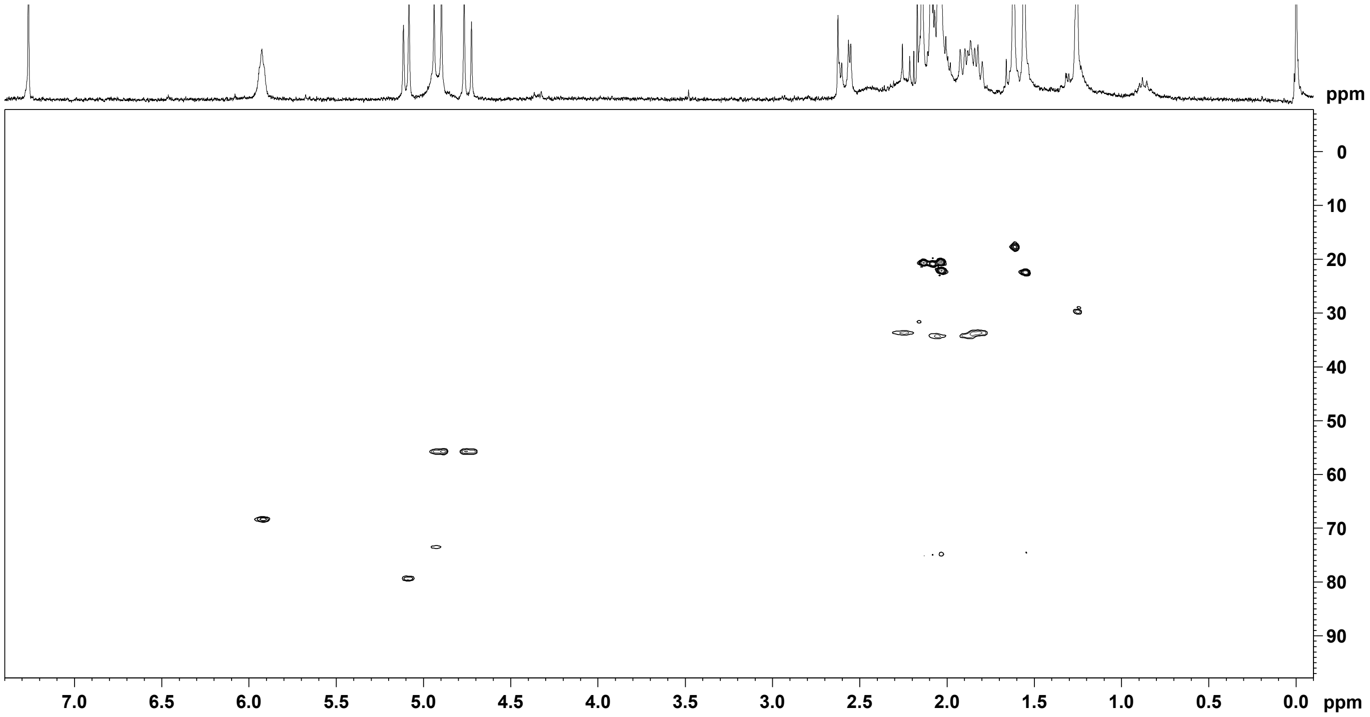

Chemical shift, in ppm (referenced to TMS - δ 0.00).

Figure S42 – HMBC correlation map ($CDCl_3$, 308 K) of semisynthetic derivative **7a**.

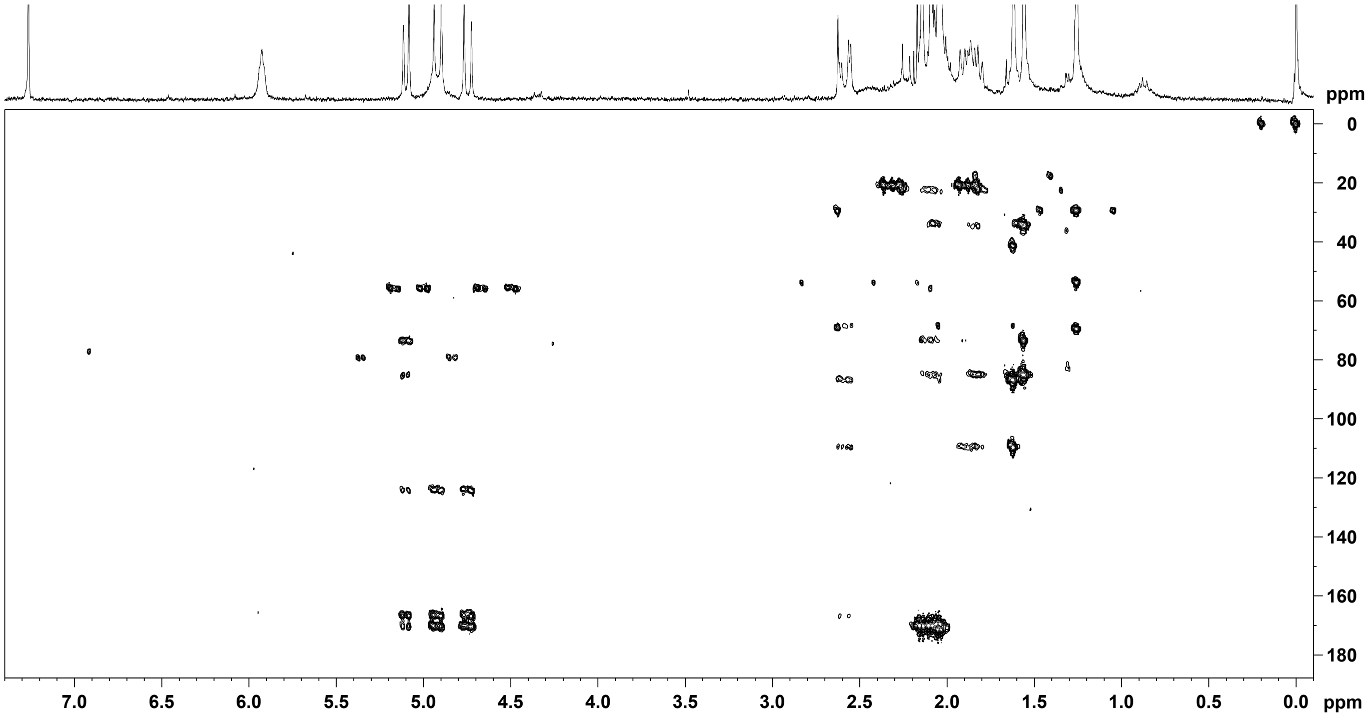

Chemical shift, in ppm (referenced to TMS - δ 0.00).

Figure S43 – HRESIMS *full scan* (100-1200 Da) spectrum of semisynthetic derivative **7a**.

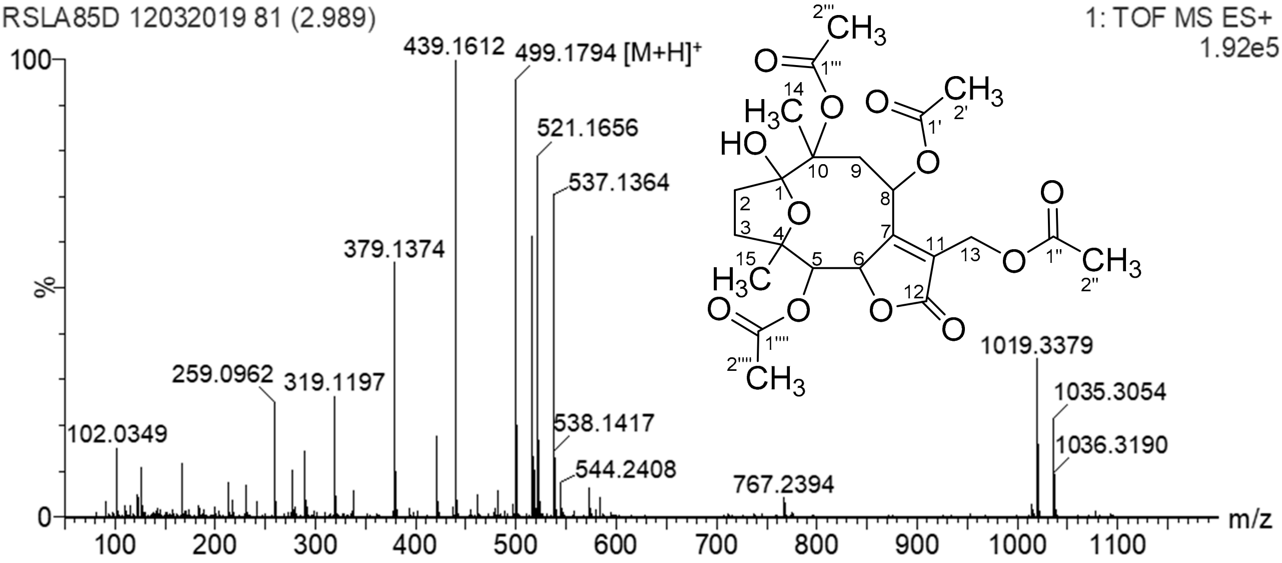

HRESIMS spectrum in positive mode achieved from UPLC-MS analysis.

Figure S44 – UV spectrum (200-400 nm) of semisynthetic derivative **7a**.

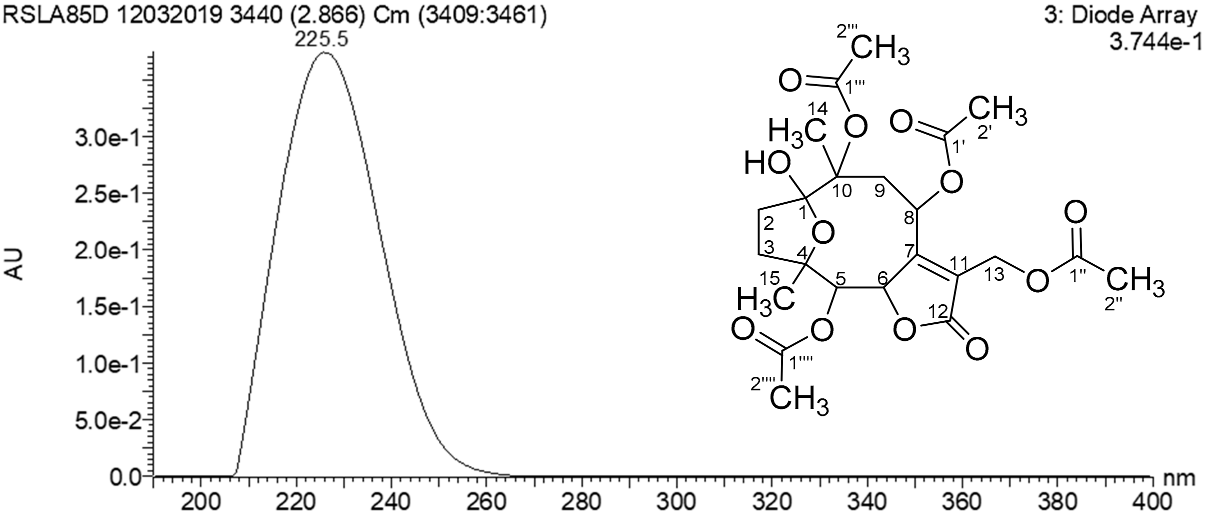

UV spectrum achieved from UPLC-PDA-MS analysis.

Figure S45 – ^{1}H NMR spectrum (300 MHz, $CDCl_3$, 295 K) of semisynthetic derivative **8a**.

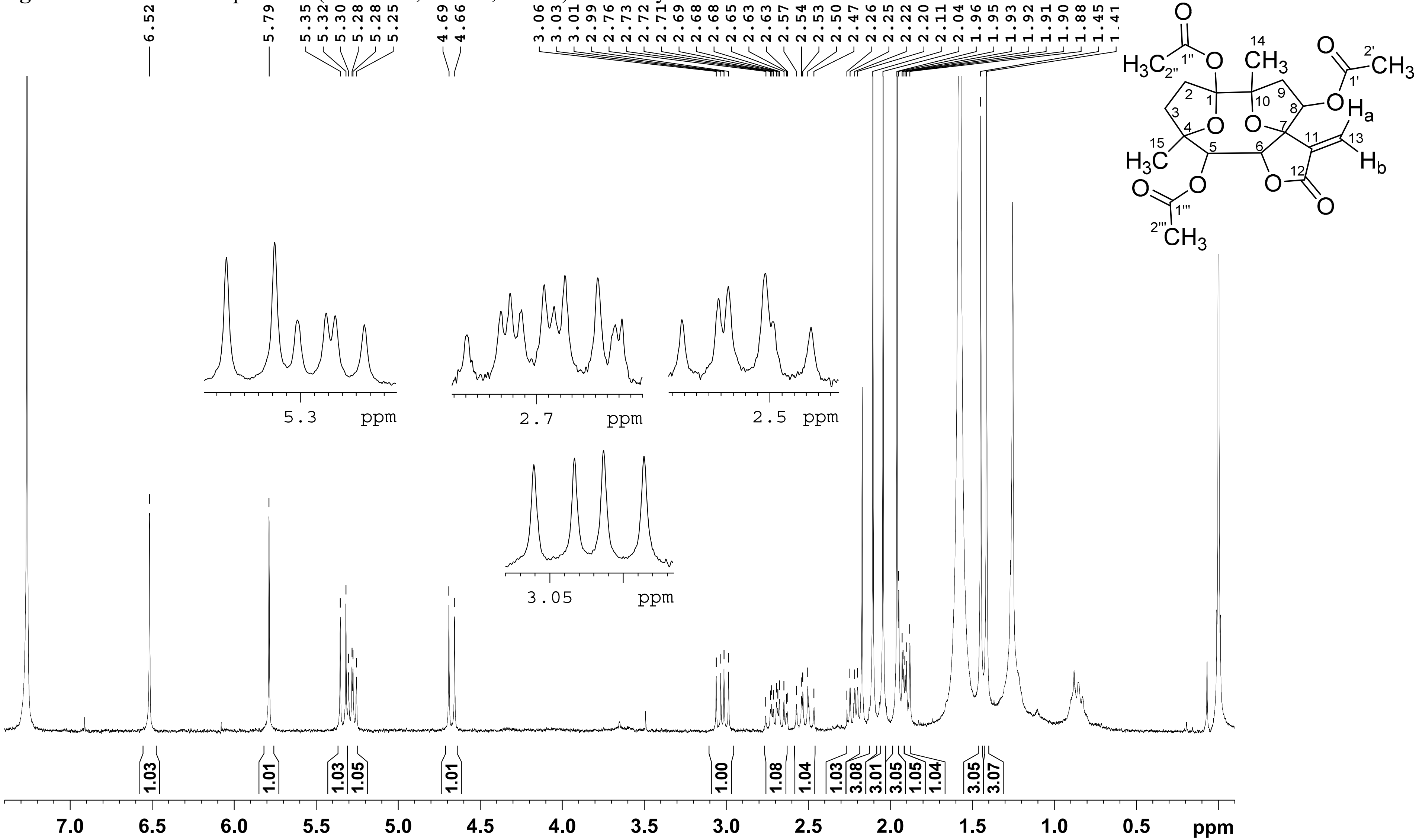

Chemical shift, in ppm (referenced to TMS - δ 0.00).

Figure S46 – HSQC correlation map ($CDCl_3$, 295 K) of semisynthetic derivative **8a**.

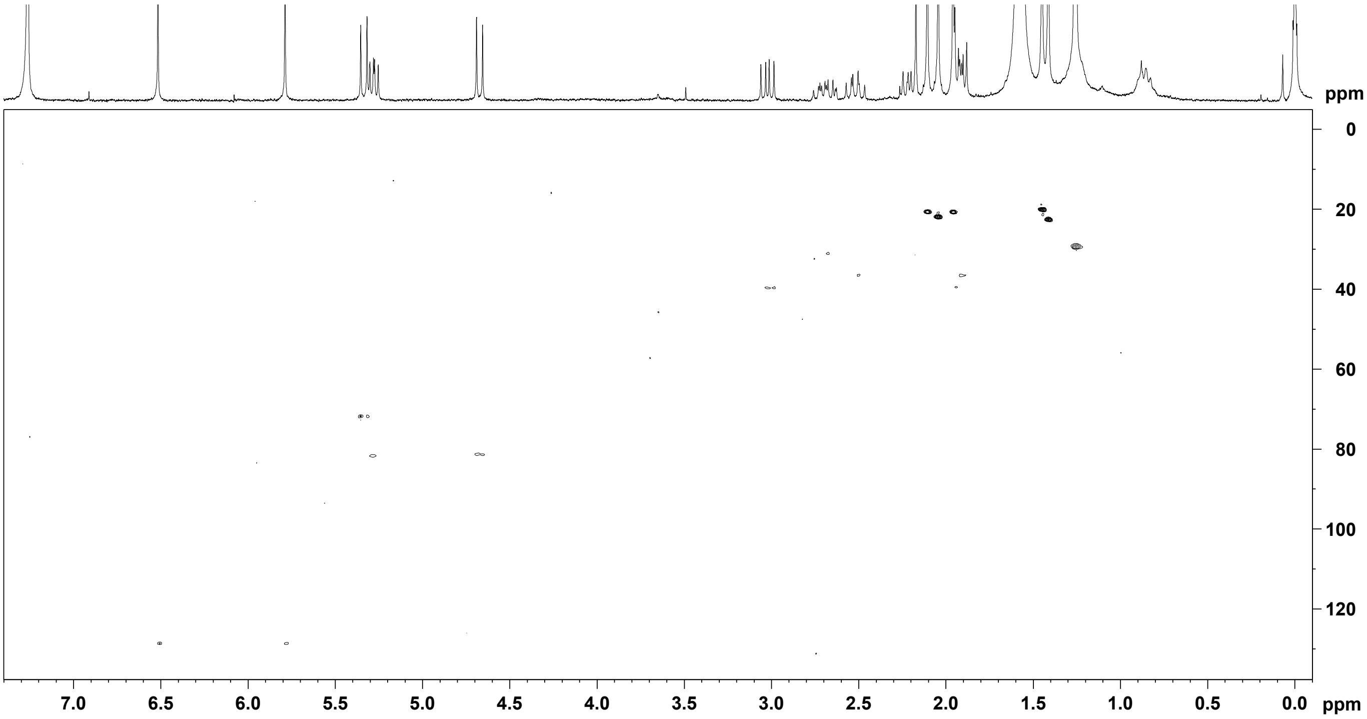

Chemical shift, in ppm (referenced to TMS - δ 0.00).

Figure S47 – HMBC correlation map ($CDCl_3$, 295 K) of semisynthetic derivative **8a**.

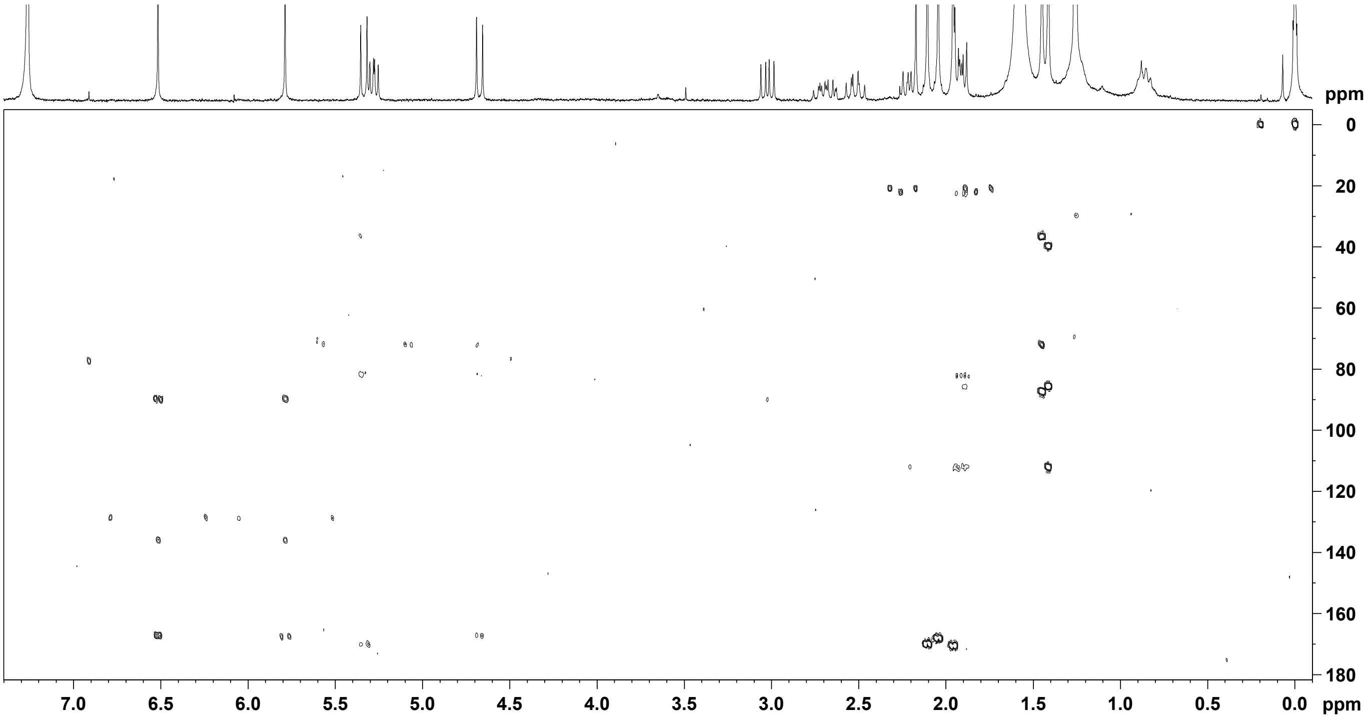

Chemical shift, in ppm (referenced to TMS - δ 0.00).

Figure S48 – HRESIMS *full scan* (100-1200 Da) spectrum of semisynthetic derivative **8a**.

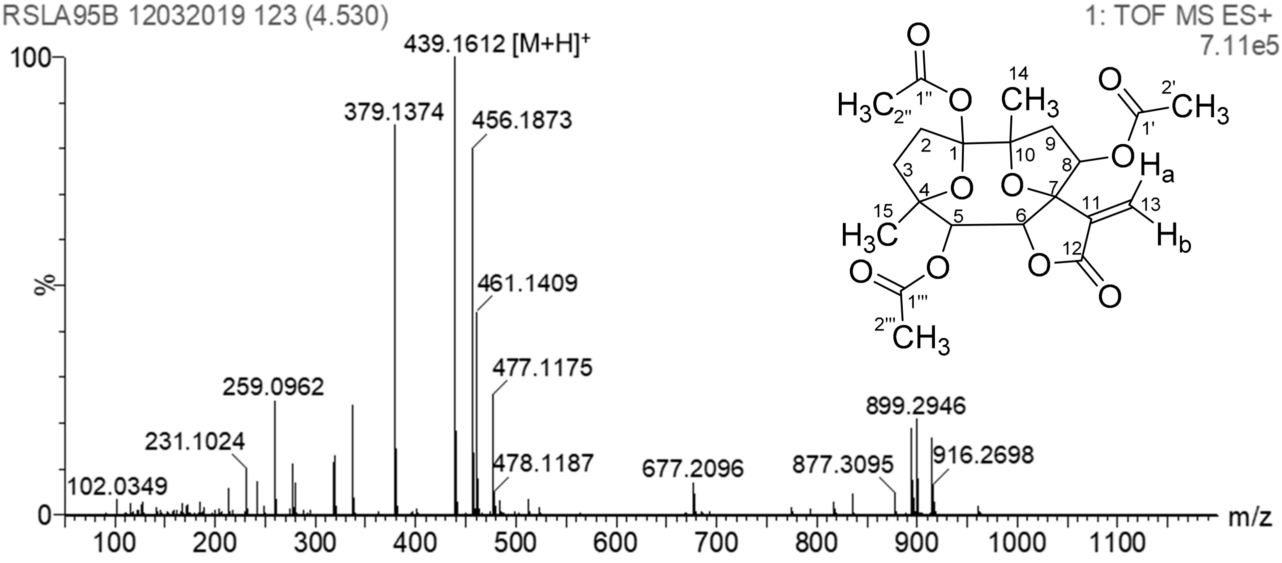

HRESIMS spectrum in positive mode achieved from UPLC-MS analysis.

Figure S49 – UV spectrum (200-400 nm) of semisynthetic derivative **8a**.

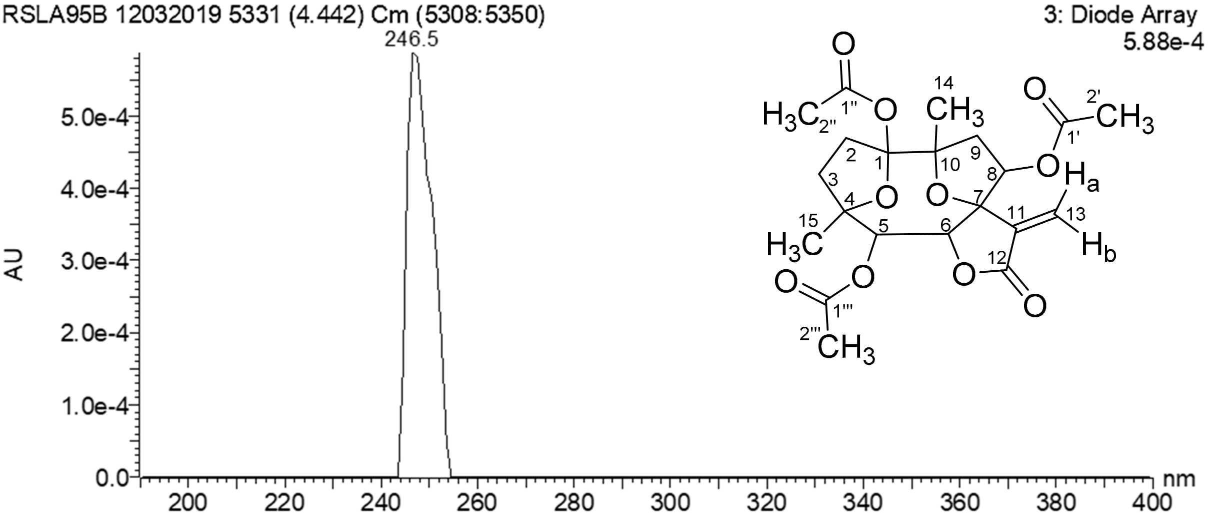

UV spectrum achieved from UPLC-PDA-MS analysis.

Figure S50 – ^{1}H NMR spectrum (300 MHz, $CDCl_3$, 295 K) of semisynthetic derivative **9a**.

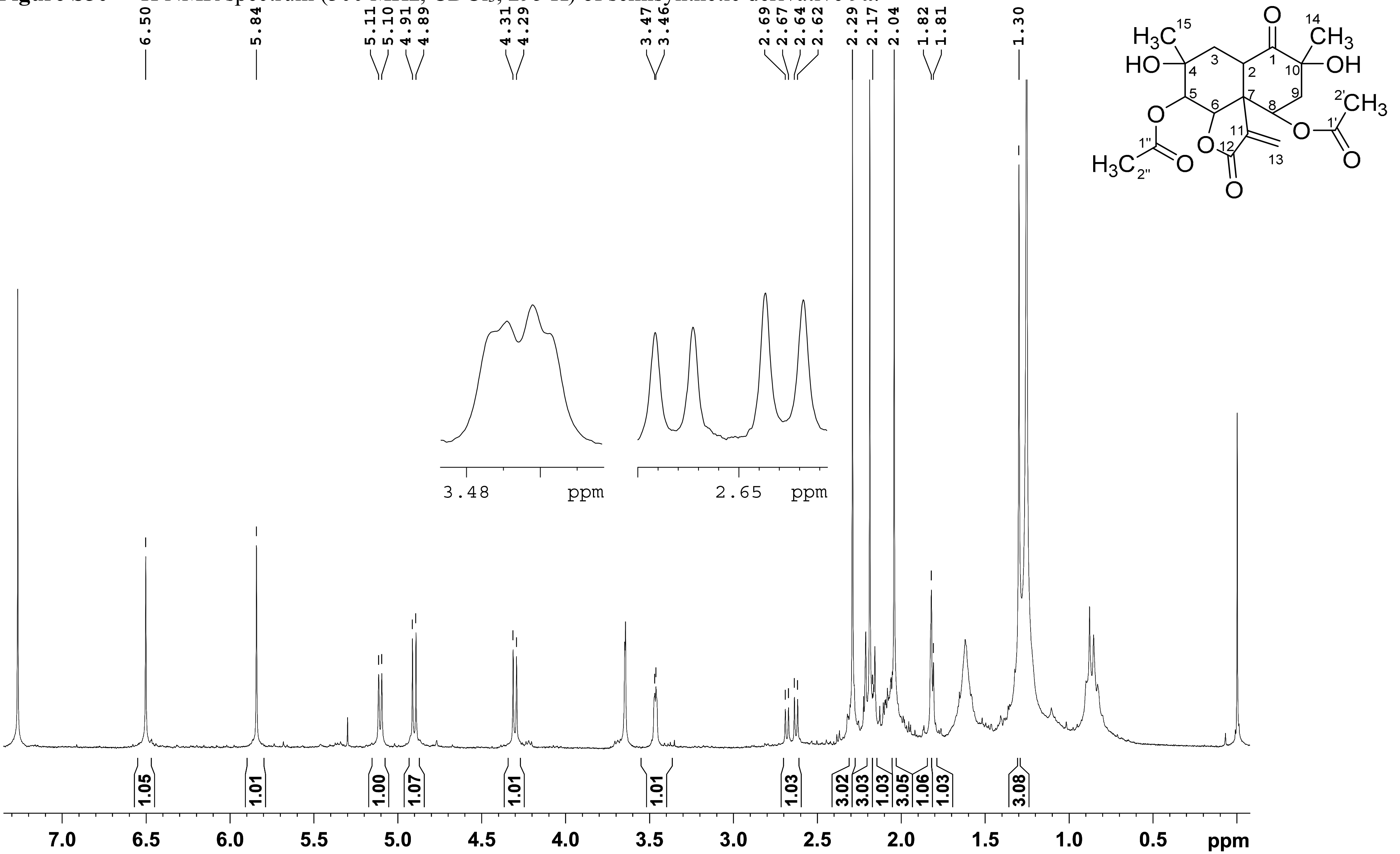

Chemical shift, in ppm (referenced to TMS - δ 0.00).

Figure S51 – HSQC correlation map ($CDCl_3$, 295 K) of semisynthetic derivative **9a**.

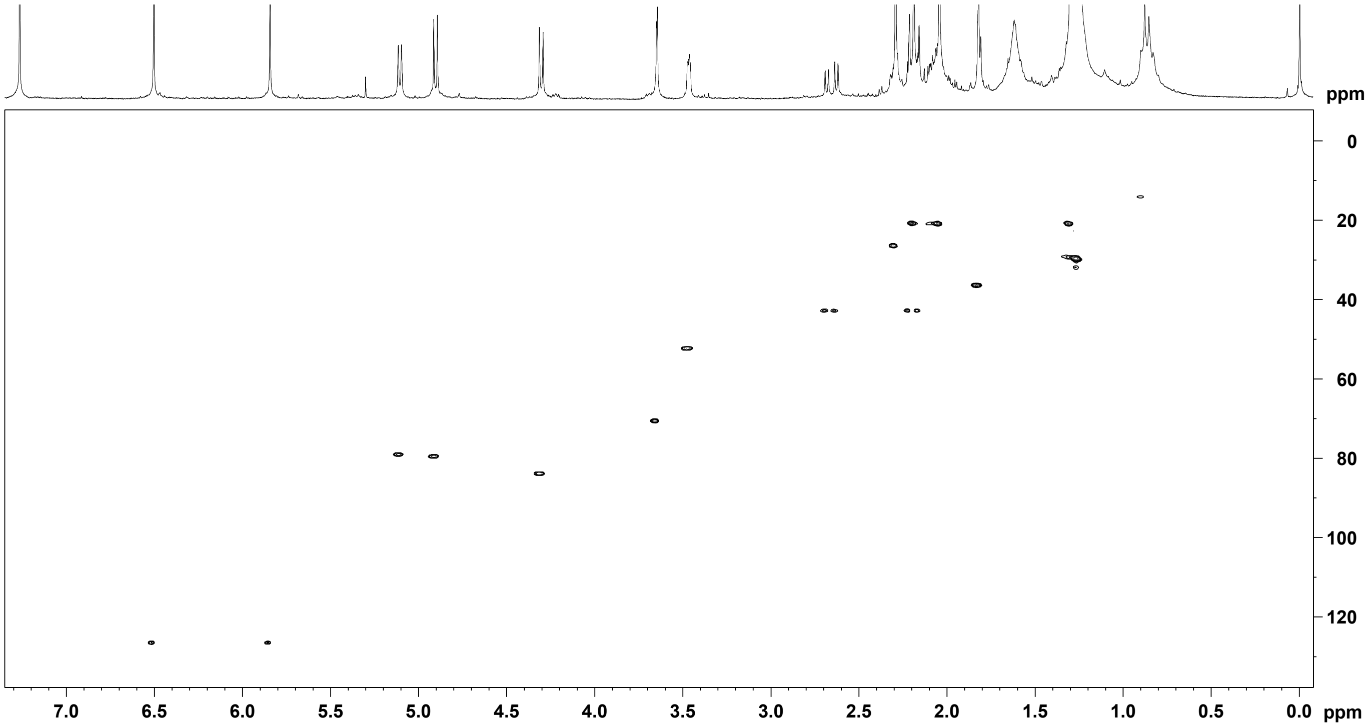

Chemical shift, in ppm (referenced to TMS - δ 0.00).

Figure S52 – HMBC correlation map ($CDCl_3$, 295 K) of semisynthetic derivative **9a**.

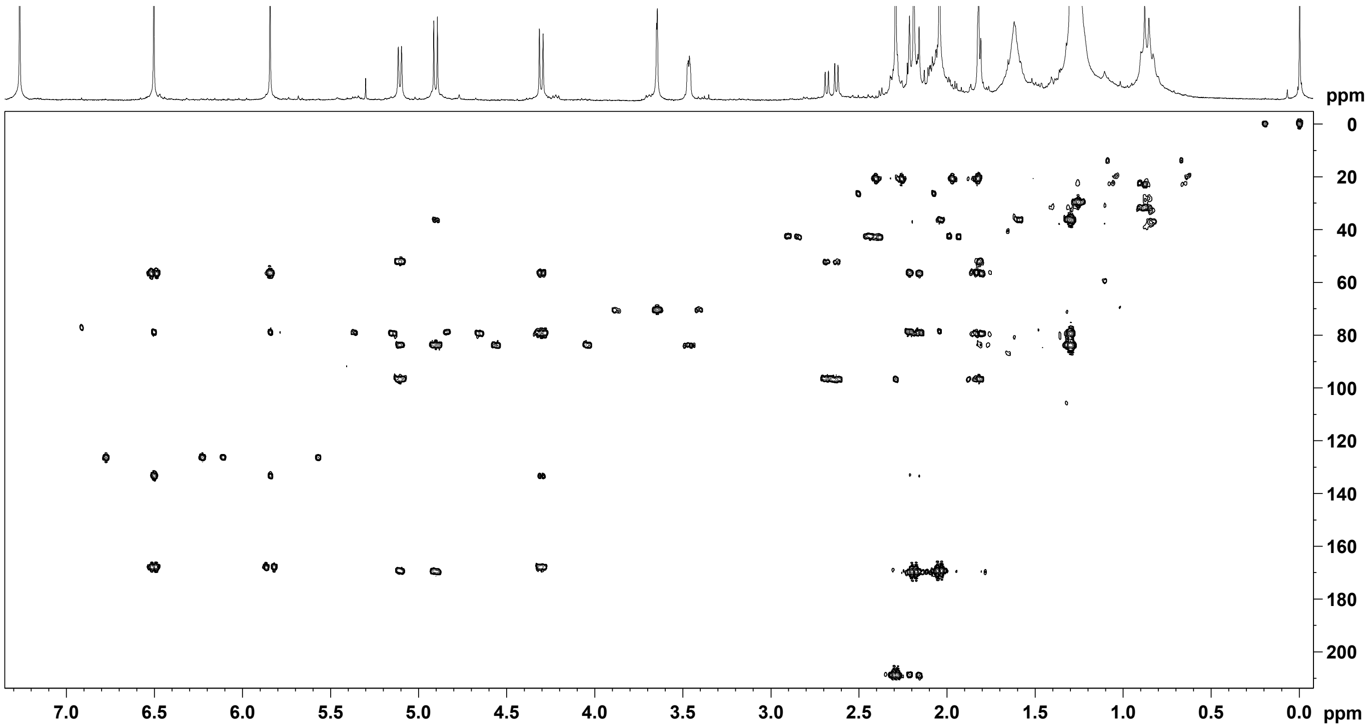

Chemical shift, in ppm (referenced to TMS - δ 0.00).

Figure S53 – HRESIMS *full scan* (100-1200 Da) spectrum of semisynthetic derivative **9a**.

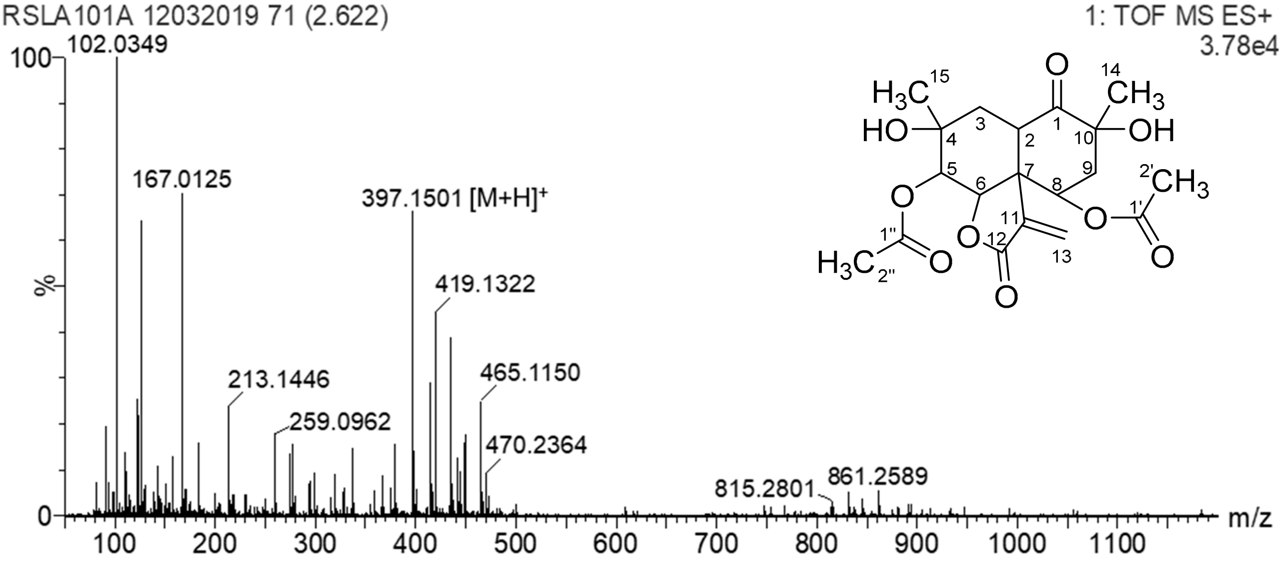

HRESIMS spectrum in positive mode achieved from UPLC-MS analysis.

Figure S54 – UV spectrum (200-400 nm) of semisynthetic derivative **9a**.

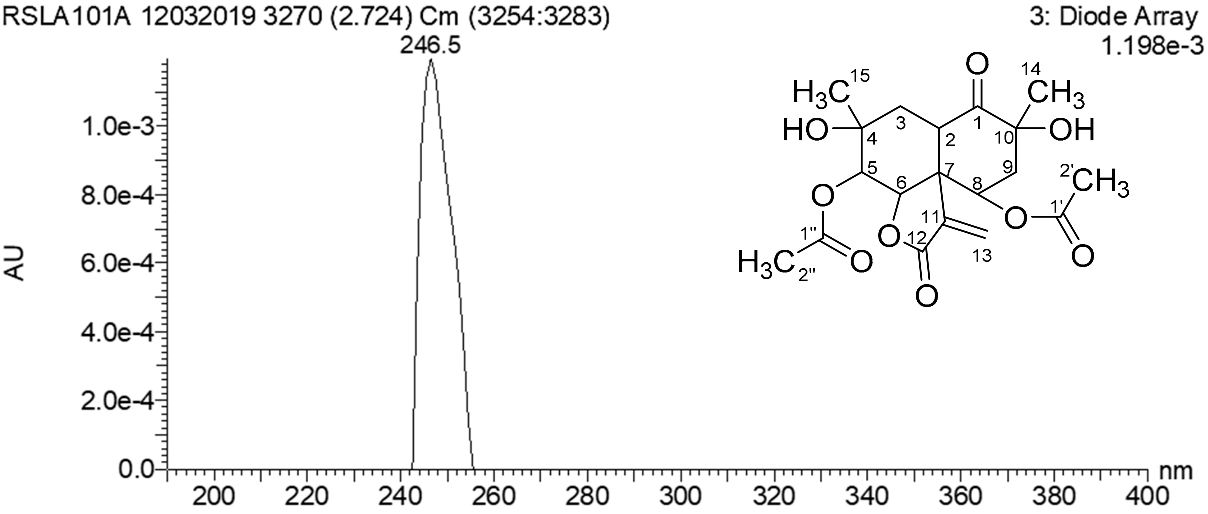

UV spectrum achieved from UPLC-PDA-MS analysis.

Figure S55 – ^{1}H NMR spectrum (300 MHz, $CDCl_3$, 295 K) of semisynthetic derivative **7b**.

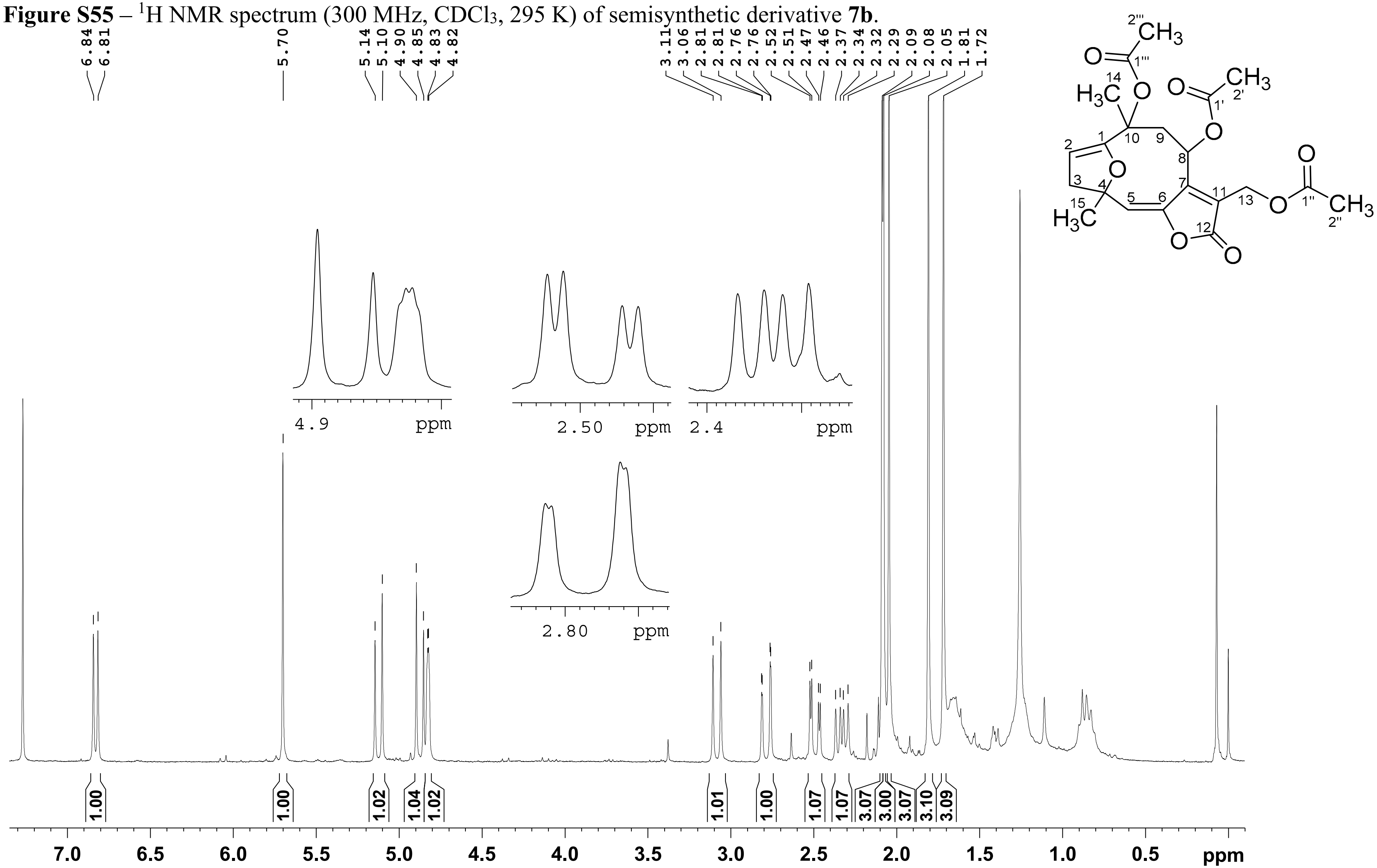

Chemical shift, in ppm (referenced to TMS - δ 0.00).

Figure S56 – HSQC correlation map ($CDCl_3$, 295 K) of semisynthetic derivative **7b**.

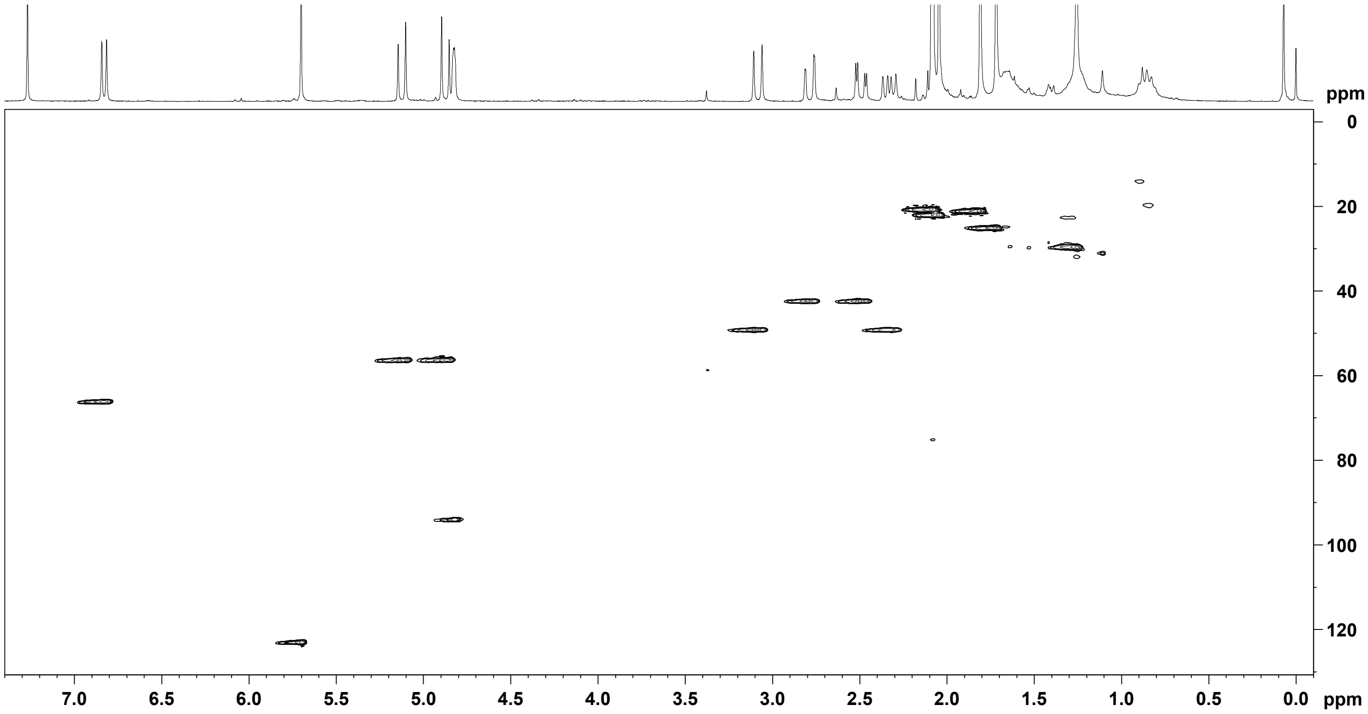

Chemical shift, in ppm (referenced to TMS - δ 0.00).

Figure S57 – HMBC correlation map ($CDCl_3$, 295 K) of semisynthetic derivative **7b**.

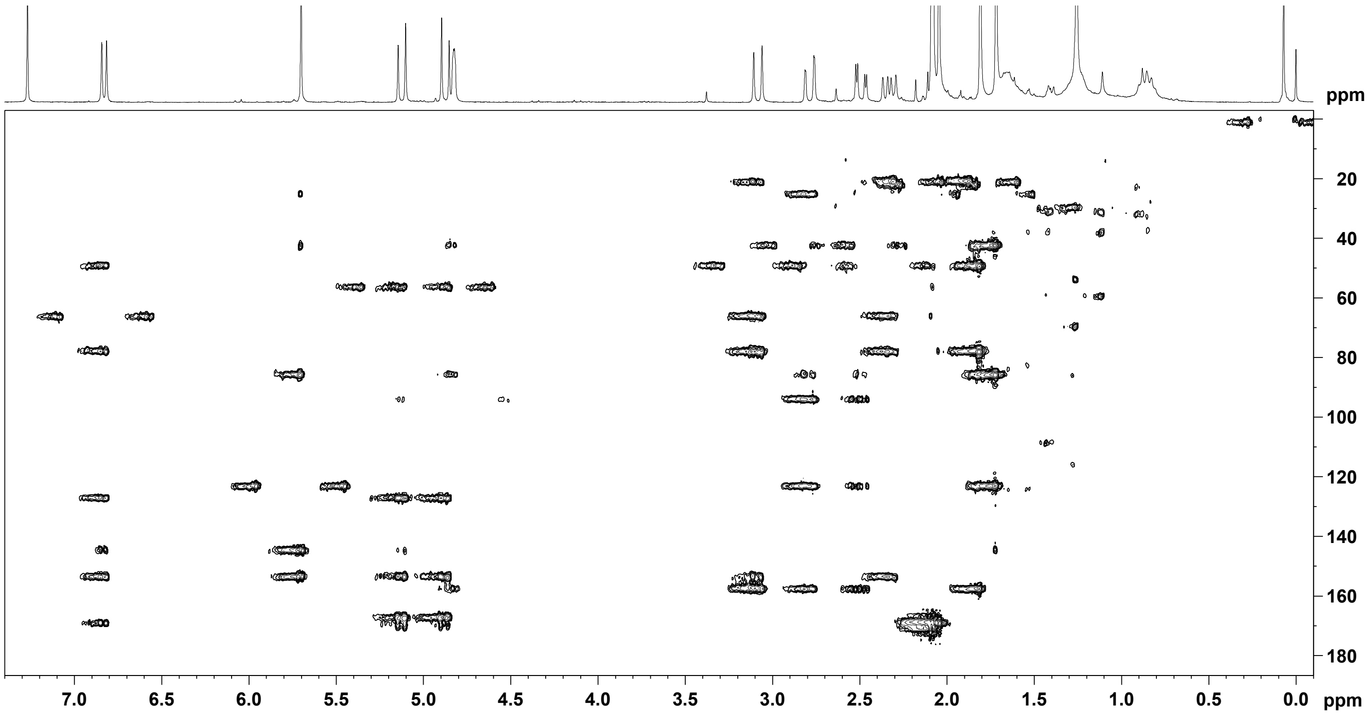

Chemical shift, in ppm (referenced to TMS - δ 0.00).

Figure S58 – HRESIMS *full scan* (100-1200 Da) spectrum of semisynthetic derivative **7b**.

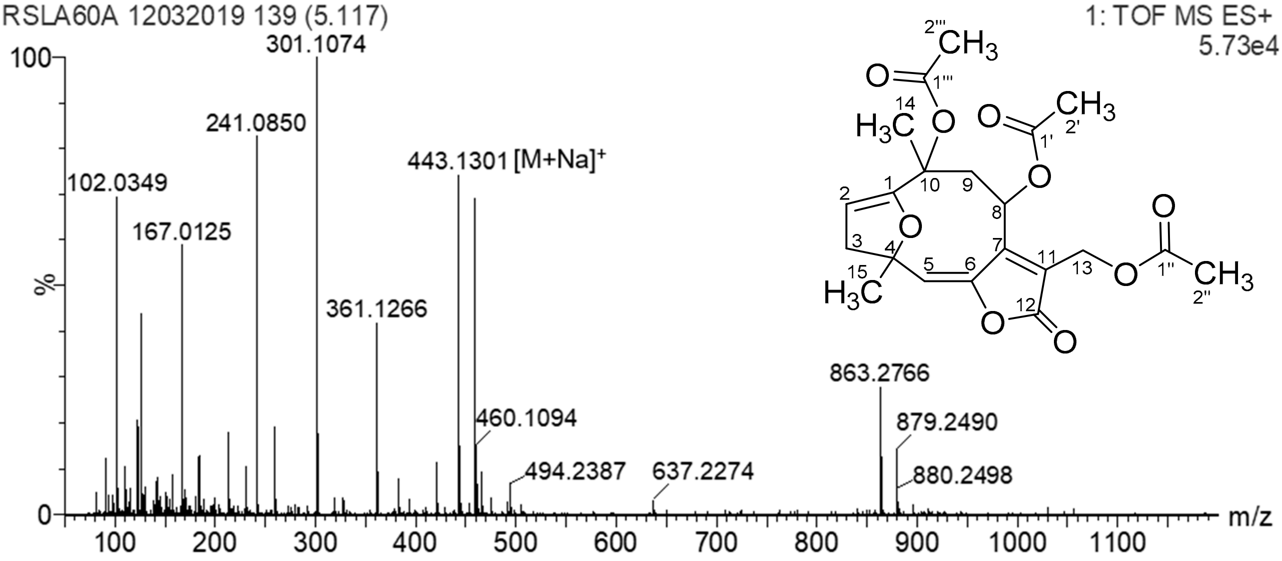

HRESIMS spectrum in positive mode achieved from UPLC-MS analysis.

Figure S59 – UV spectrum (200-400 nm) of semisynthetic derivative **7b**.

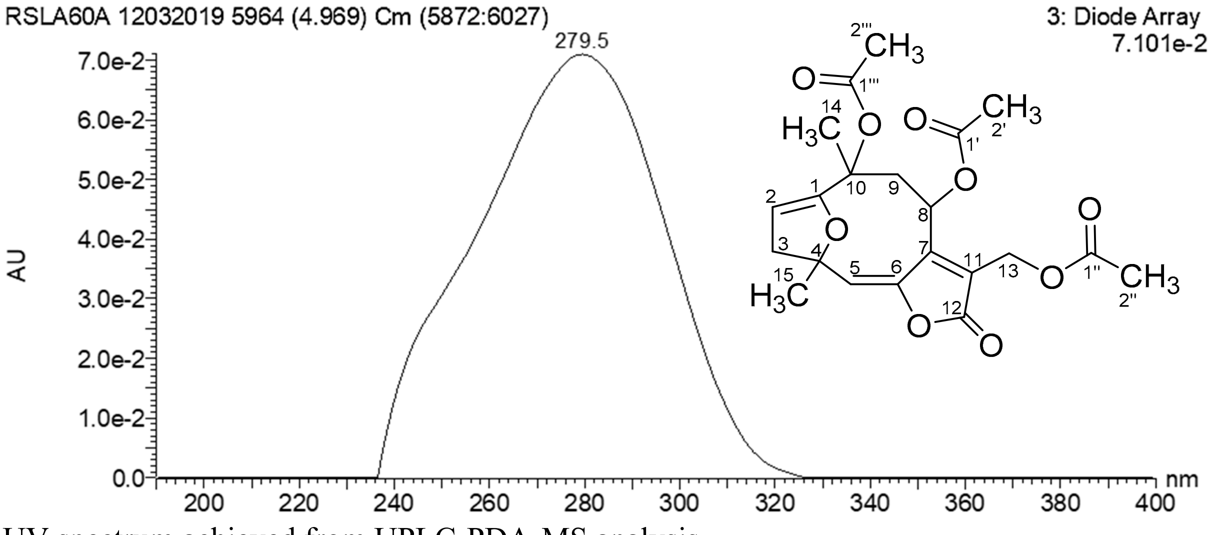

UV spectrum achieved from UPLC-PDA-MS analysis.

Figure S60 – ^{1}H NMR spectrum (300 MHz, $CDCl_3$, 278 K) of semisynthetic derivative **7c**.

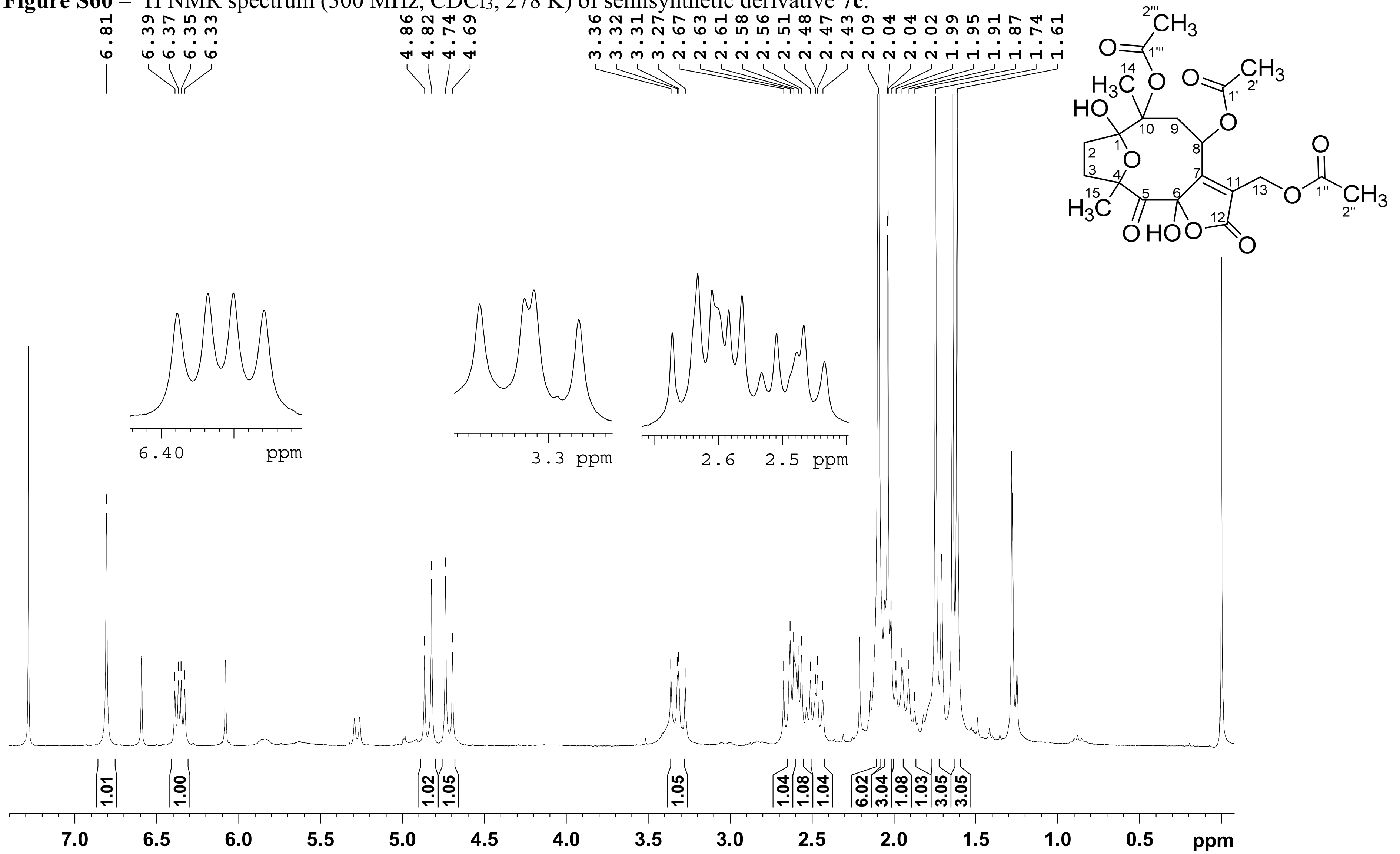

Chemical shift, in ppm (referenced to TMS - δ 0.00).

Figure S61 – HSQC correlation map ($CDCl_3$, 278 K) of semisynthetic derivative **7c**.

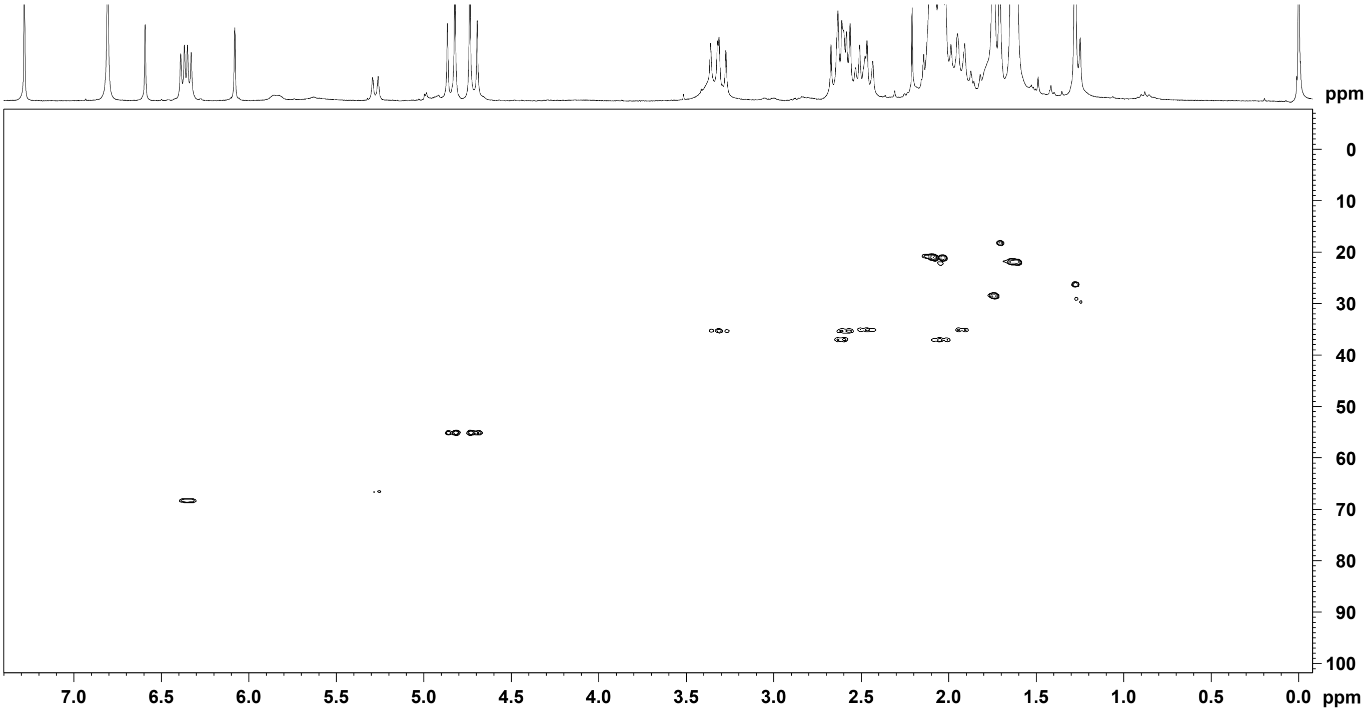

Chemical shift, in ppm (referenced to TMS - δ 0.00).

Figure S62 – HMBC correlation map ($CDCl_3$, 278 K) of semisynthetic derivative **7c**.

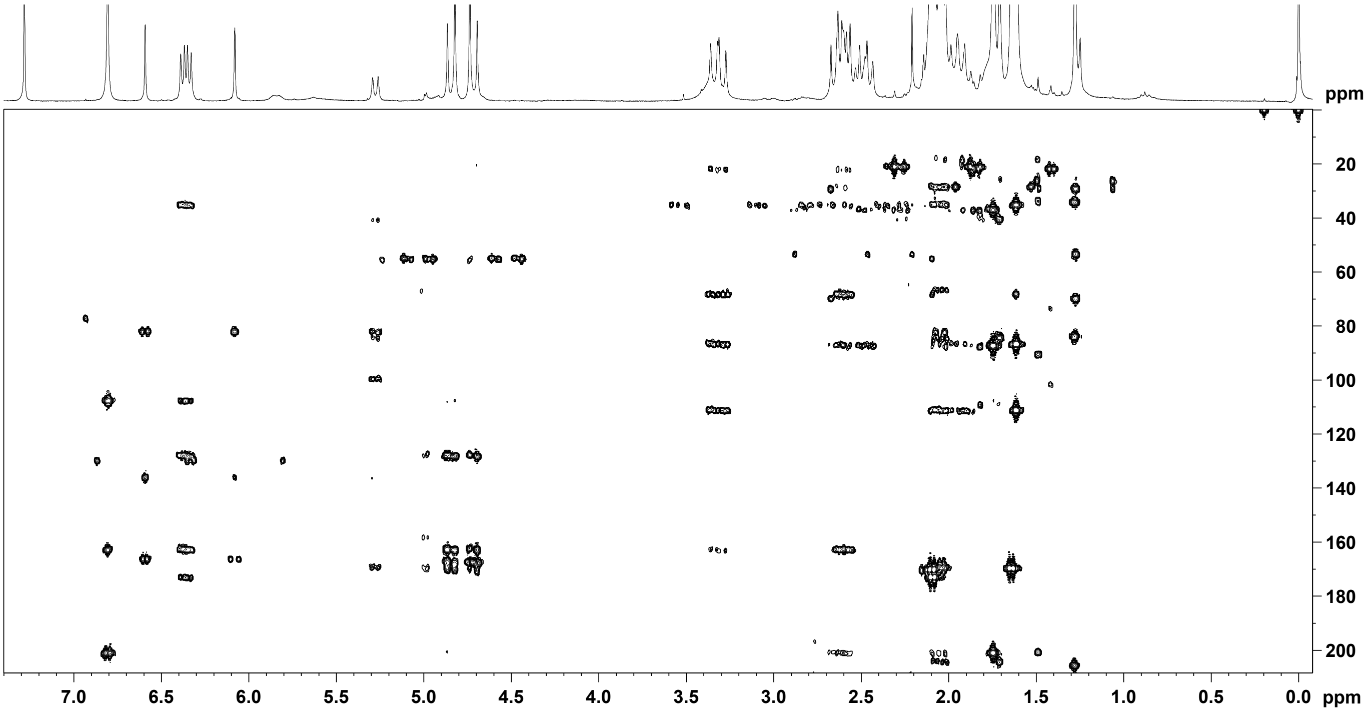

Chemical shift, in ppm (referenced to TMS - δ 0.00).

Figure S63 – HRESIMS *full scan* (100-1200 Da) spectrum of semisynthetic derivative **7c**.

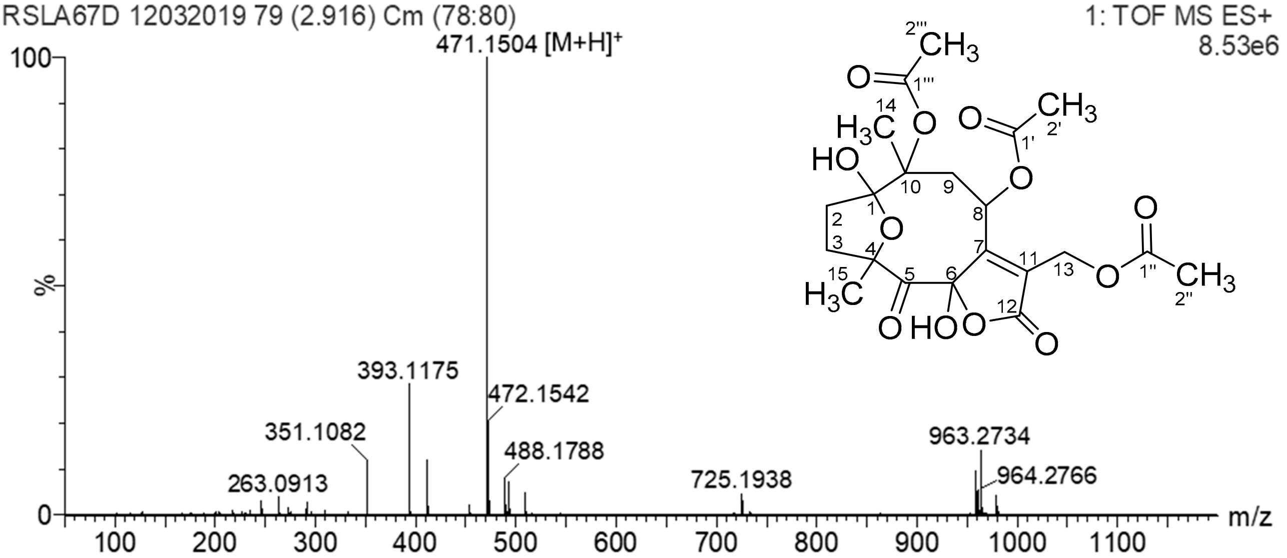

HRESIMS spectrum in positive mode achieved from UPLC-MS analysis.

Figure S64 – UV spectrum (200-400 nm) of semisynthetic derivative **7c**.

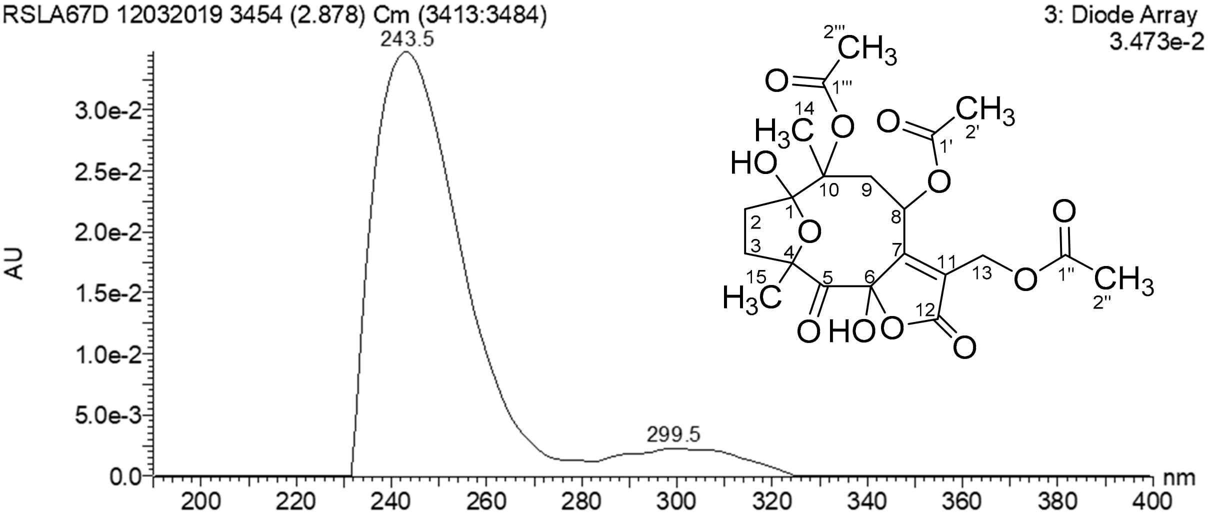

UV spectrum achieved from UPLC-PDA-MS analysis.

Figure S65 – ^{1}H NMR spectrum (300 MHz, $CDCl_3$, 295 K) of semisynthetic derivative **7c.1**.

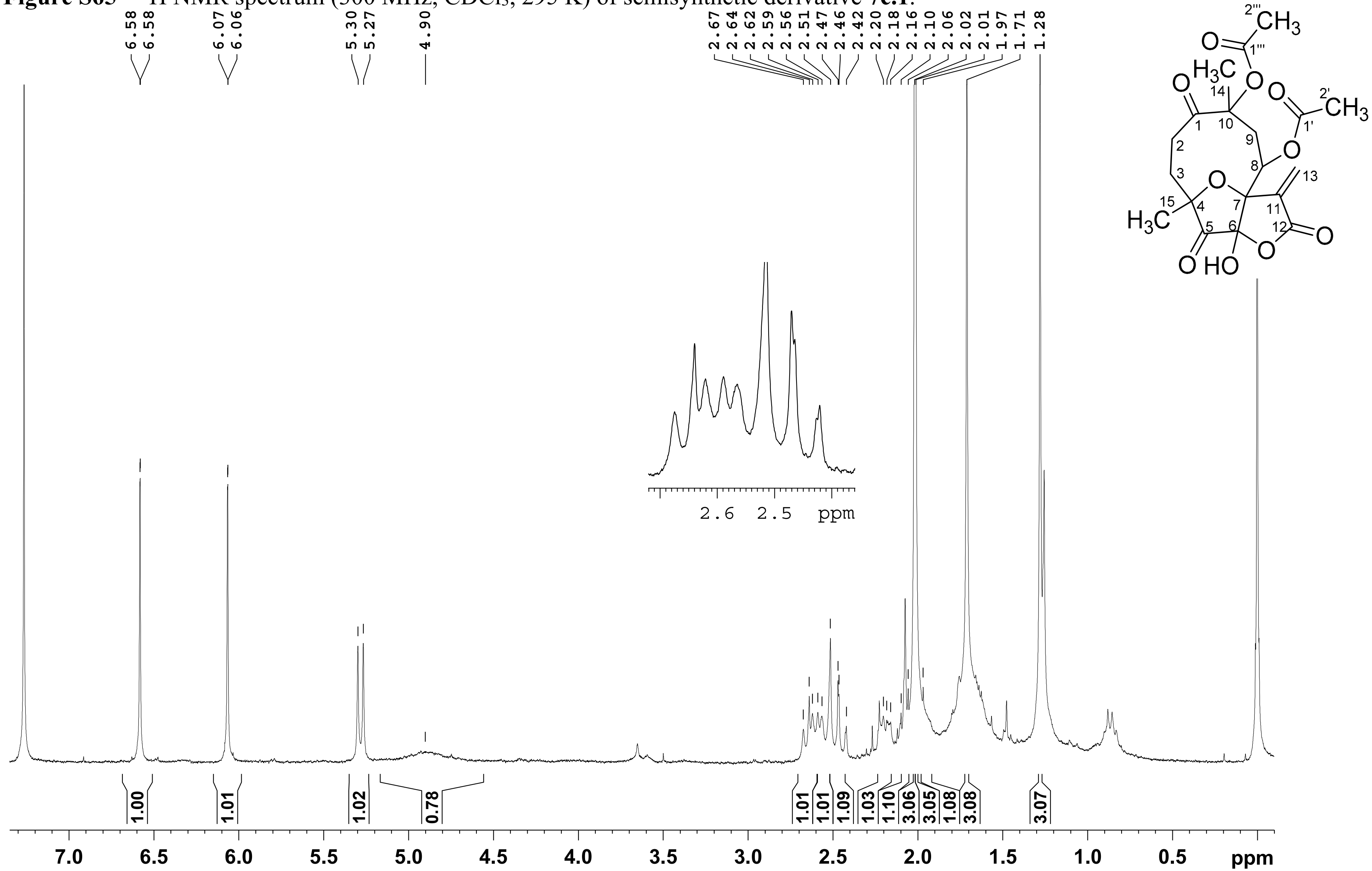

Chemical shift, in ppm (referenced to TMS - δ 0.00).

Figure S66 – HSQC correlation map ($CDCl_3$, 295 K) of semisynthetic derivative **7c.1**.

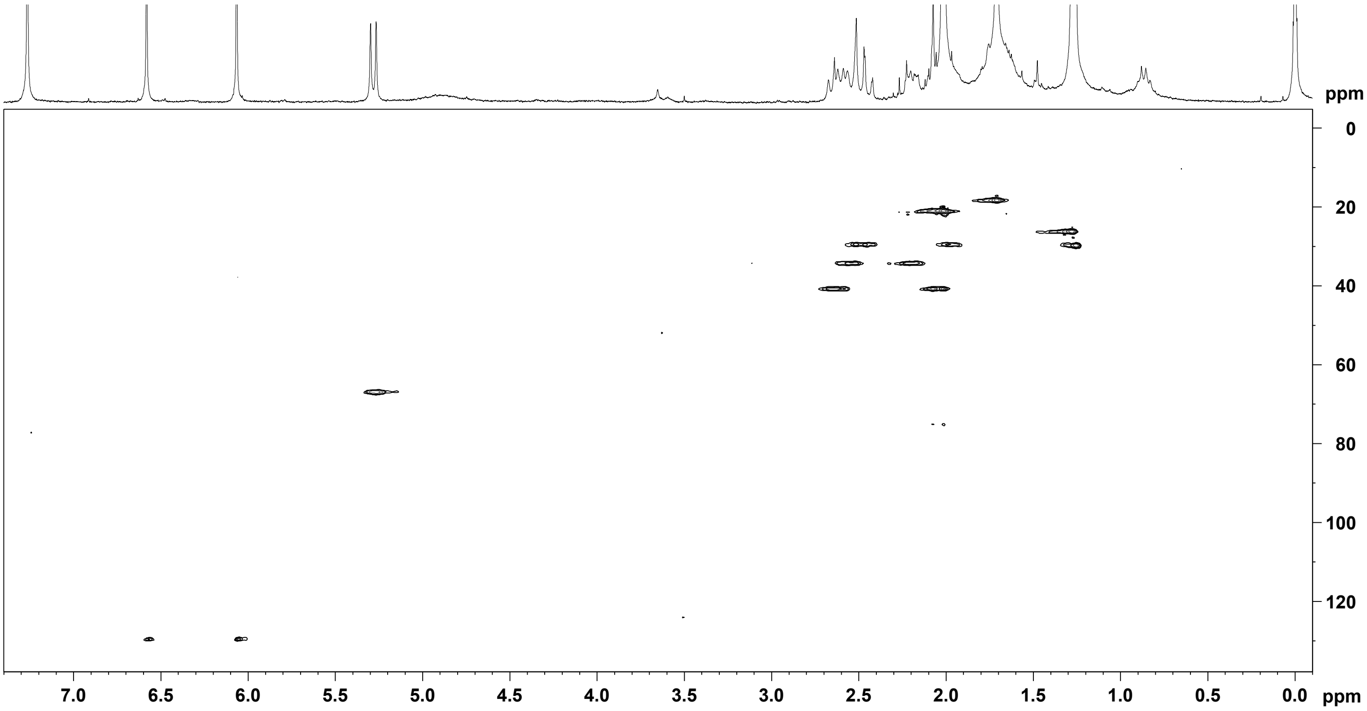

Chemical shift, in ppm (referenced to TMS - δ 0.00).

Figure S67 – HMBC correlation map ($CDCl_3$, 295 K) of semisynthetic derivative **7c.1**.

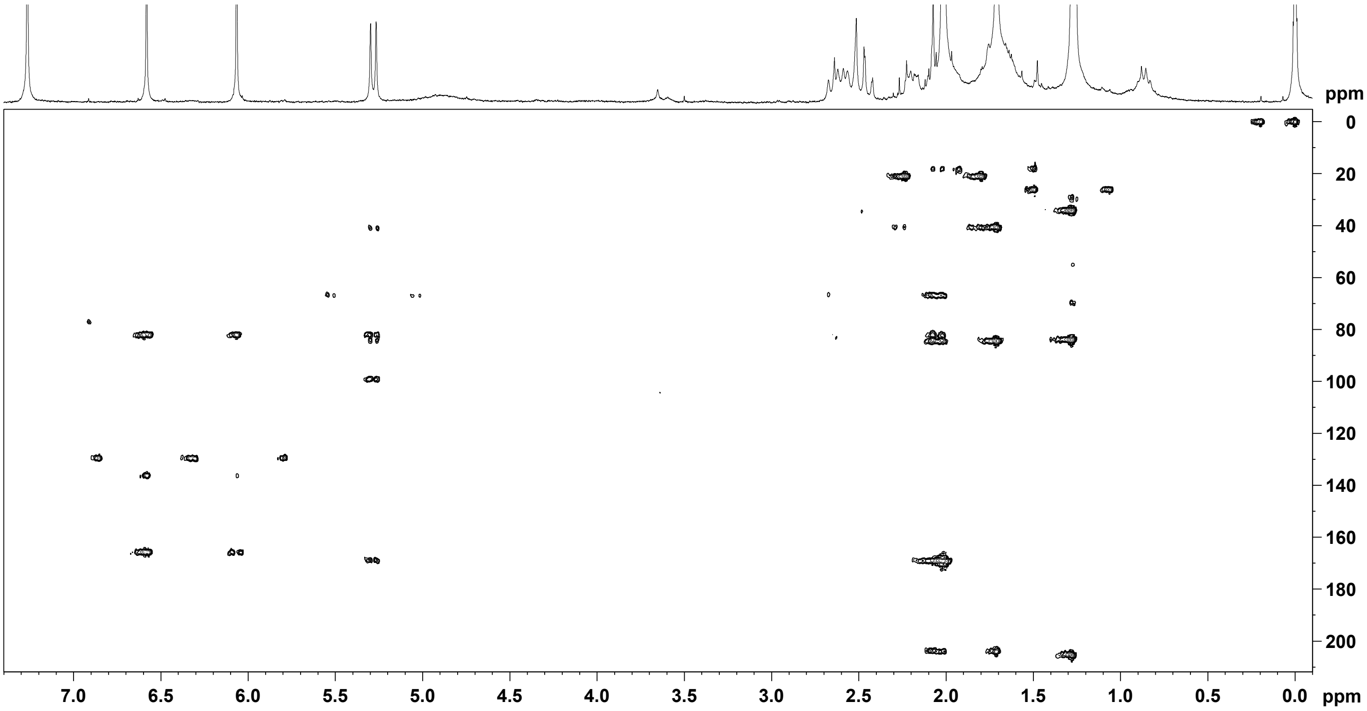

Chemical shift, in ppm (referenced to TMS - δ 0.00).

Figure S68 – HRESIMS *full scan* (100-1200 Da) spectrum of semisynthetic derivative **7c.1**.

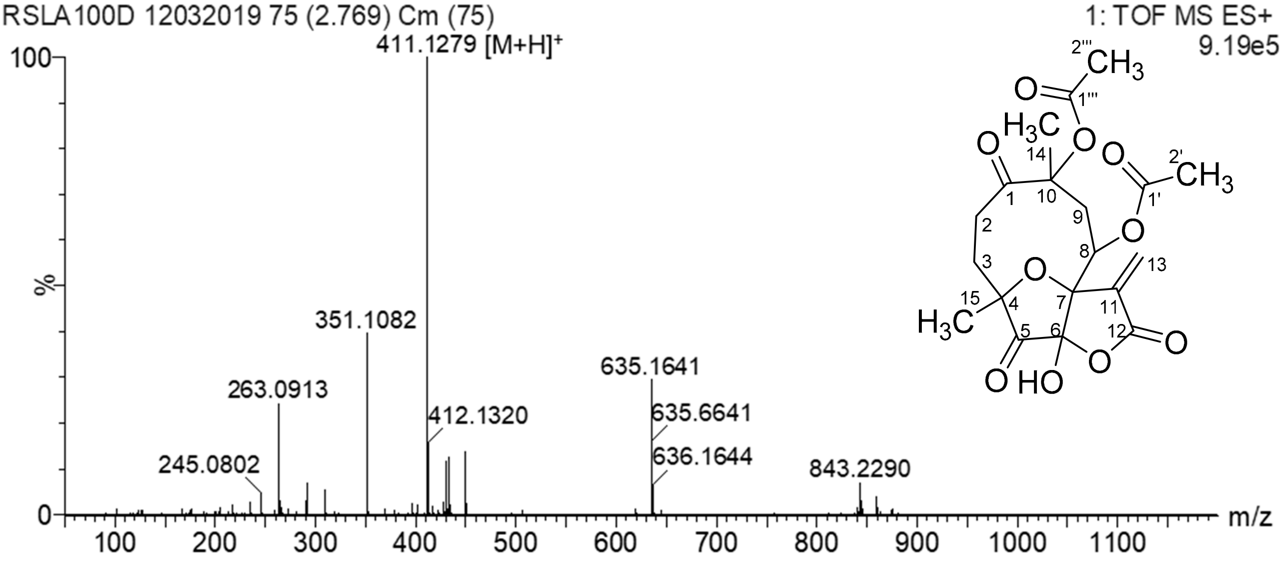

HRESIMS spectrum in positive mode achieved from UPLC-MS analysis.

Figure S69 – UV spectrum (200-400 nm) of semisynthetic derivative **7c.1**.

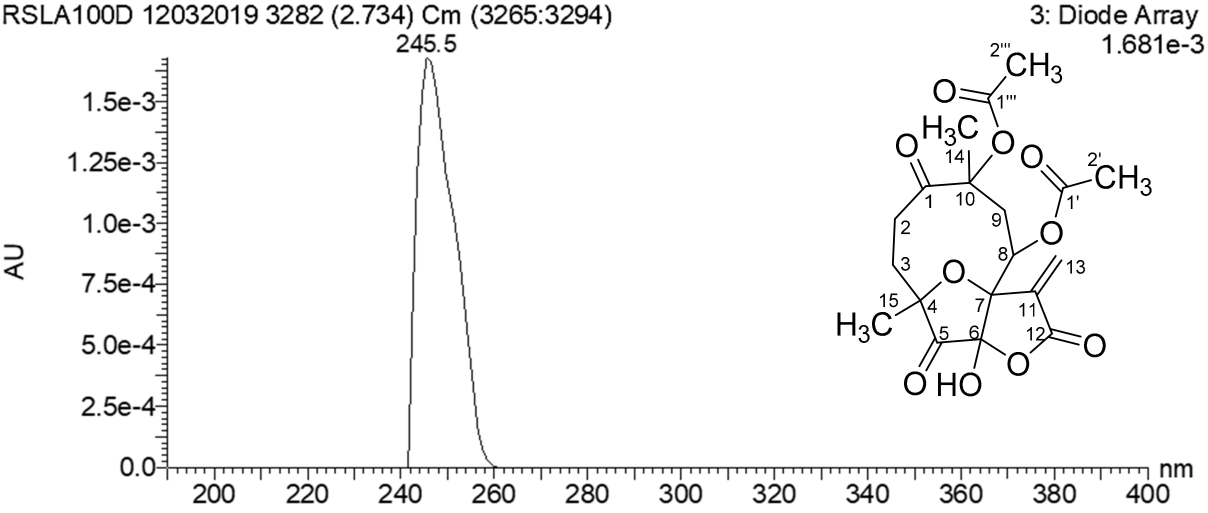

UV spectrum achieved from UPLC-PDA-MS analysis.

Figure S70 – Evaluation of the macrophage J774A.1 cell viability treated with SLs **1-12**.

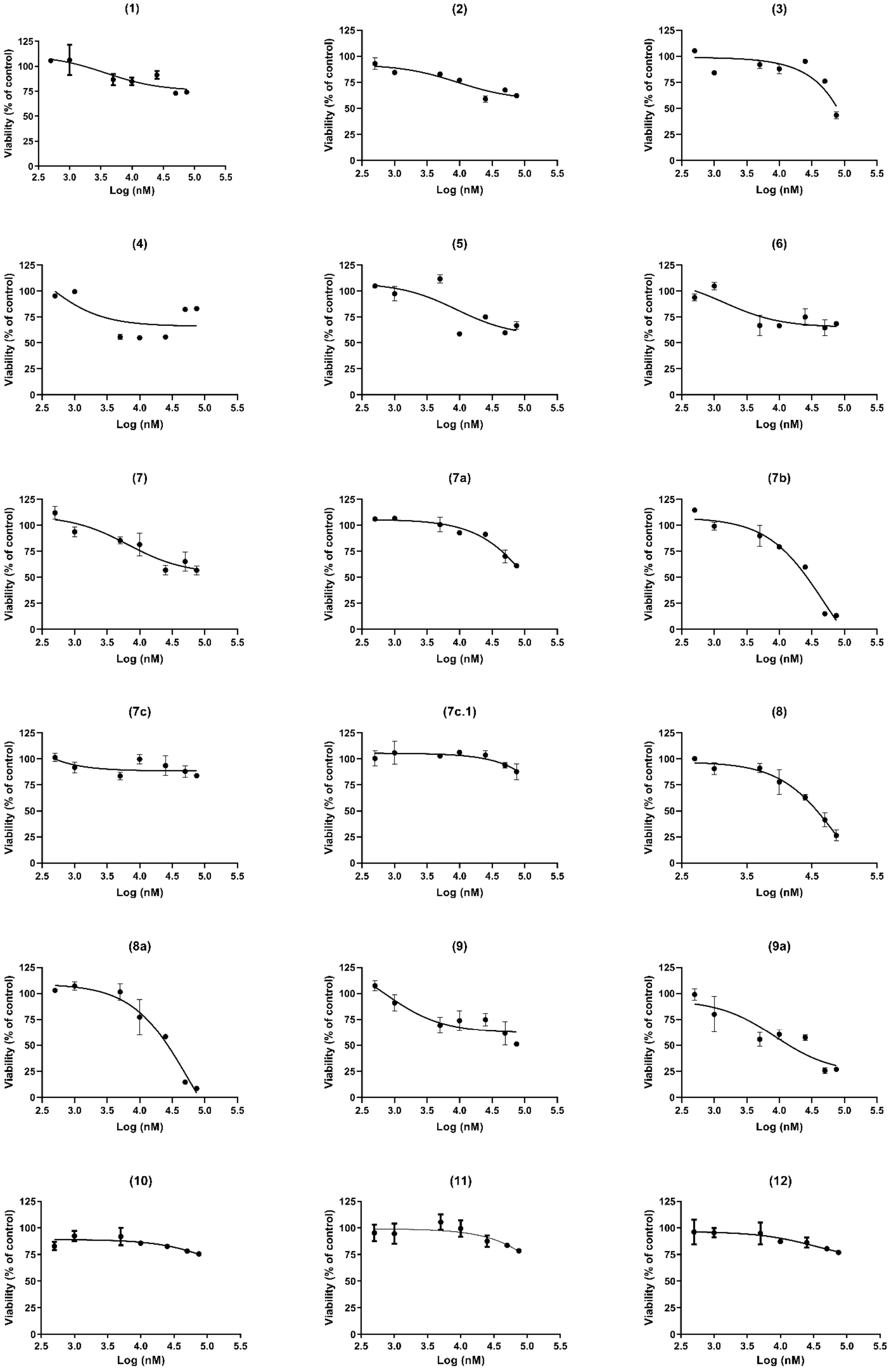

Supplement: Supplementary file 1 [file molecules-28-01243-s001.zip › molecules-2154396-supplementary.pdf]
